# Measuring mobility in HIV research in sub‐Saharan Africa: a scoping review

**DOI:** 10.1002/jia2.26508

**Published:** 2025-06-05

**Authors:** Aleya Khalifa, Sara Wallach, M. Kate Grabowski, Dustin T. Duncan, Fred Nalugoda, Quarraisha Abdool Karim, Barun Mathema

**Affiliations:** ^1^ Department of Epidemiology Mailman School of Public Health, Columbia University New York New York USA; ^2^ ICAP at Columbia University New York New York USA; ^3^ Department of Epidemiology Bloomberg School of Public Health, Johns Hopkins University Baltimore Maryland USA; ^4^ Department of Pathology Johns Hopkins School of Medicine Baltimore Maryland USA; ^5^ Rakai Health Sciences Program Kalisizo Uganda; ^6^ Centre for the AIDS Programme of Research in South Africa (CAPRISA), University of KwaZulu‐Natal Durban South Africa

**Keywords:** HIV/AIDS, HIV care and treatment, HIV prevention, human mobility, migration, travel

## Abstract

**Introduction:**

Mobility—from overnight travel to permanent migration—can reduce service access and increase HIV risk, driving the epidemic in sub‐Saharan Africa (SSA). This scoping review described mobility measures used in HIV research to identify gaps and guide research on mobility to strengthen HIV responses in SSA.

**Methods:**

Literature from three databases (PubMed, Embase, Web of Science) were systematically screened to identify research articles examining relationships between mobility and individual‐level HIV‐related outcomes in SSA from 2014 through 2023. Key terms for mobility included “mobility,” “movement,” “migration” and “travel.” Measures were first extracted according to International Organization of Migration definitions of migration (a change in the place of usual residence) and travel (movement between geographies). Then, metrics used to categorize or quantify mobility were organized by the stage (origin, transit, destination, return) and dimension (spatial, temporal, socio‐structural) of the movement captured. Measures were analysed within three research contexts: the HIV outcome(s) of interest, study population and local geographies. Outcomes included HIV acquisition, AIDS‐related death, and indicators along the prevention, care and treatment cascade.

**Results:**

We identified 69 studies after screening 5343 titles/abstracts and 200 full texts for eligibility. Studies included research from 16 countries, mostly representing general adult populations in eastern and southern Africa. Most studies measured migration (51) versus travel (21) and examined relationships with HIV prevalent infection (29) or care and treatment indicators (44) compared to other epidemiological and programmatic outcomes. Studies employed a range of metrics, mostly of the duration of stay at the destination (28), the number of mobility events (12) or the geographic boundaries across which individuals moved (14). Socio‐structural dimensions like the motivation for movement were measured less often. Only 15 studies examined more than one dimension.

**Discussion:**

Mobility measures varied widely and were inconsistently studied across research contexts. Future studies should fill evidence gaps, standardize reporting and develop multidimensional mobility measures tailored to local settings and HIV outcomes.

**Conclusions:**

People on the move are a vast and diverse group, yet they are often labelled as a monolith. Improved measures can disentangle how different forms of mobility relate to HIV, generating actionable evidence to enhance HIV programming for ending the epidemic.

## 1 INTRODUCTION

HIV incidence is on the decline in sub‐Saharan Africa (SSA) [1], but remains high. Rising population mobility could reverse these gains as people on the move experience barriers to prevention, care and treatment services, as well as elevated rates of HIV acquisition and viraemia [2, 3, 4, 5, 6]. People may also migrate or travel following an HIV diagnosis [7, 8, 9, 10]. Since mobility, or the movement of persons across geographies, can bridge otherwise unlinked sexual networks [11, 12, 13], mobile populations may sustain or amplify population‐level HIV incidence [14].

In the same years that SSA is approaching epidemic control, it is facing unprecedented rates of mobility, with over half of some countries' populations migrating internally [15]. Complex social, economic, political and environmental forces drive movement in SSA, resulting in dynamic labour‐related travel, record urbanization rates (by 2030, half of Africans will live in cities), and tens of millions of climate‐displaced people projected over the next decades [16, 17, 18, 19, 20]. Mobility will be, and has been, key for securing livelihoods and economic development [21]. For example, circular labour migration—a well‐documented driver of HIV transmission—has been essential for anyone seeking work in apartheid and post‐apartheid South Africa [22]. Hence, migrants remain a high‐risk priority population in many national HIV responses [23]. Notwithstanding, health systems remain ill‐adapted to serve the needs of people on the move [24].

Recent evidence suggests that different forms of mobility—from local trade‐related travel to cross‐border migration—may impact HIV outcomes differently [6, 25], necessitating comprehensive mobility measures to better understand the relationship between mobility and HIV. Broad definitions of mobility and programming for broad swaths of mobile populations can waste rapidly declining HIV resources. For example, young mobile men may struggle most with HIV testing and treatment adherence [26, 27], while mobile women may have higher risks of HIV acquisition than mobile men due to changing sexual partnerships during travel [28, 29, 30]. Further, each type of movement may lead to different programmatic gaps along the prevention, care and treatment cascade. Indeed, non‐work travel may cause lower treatment adherence than other forms of travel [25], and distant migrations may result in new HIV acquisitions more often than local relocations [6]. Understanding which dimensions of human movement relate to which aspects of HIV transmission risk can inform both resource allocation and tailored HIV implementation strategies for people on the move [31, 32].

While research agendas for HIV prevention [11] and treatment [33] call for robust and detailed mobility measures to inform HIV programmes, there is no unifying framework for how to select mobility metrics in relation to an HIV outcome. Such guidance is especially important in SSA where research budgets are limited, and it is unfeasible to implement long survey modules in addition to already burdensome HIV survey questionnaires. For example, some studies depend on household census data from health and demographic surveillance systems to monitor migration trends [34]. Thus, a critical first step towards designing comprehensive and standardized mobility measures is to synthesize the various ways in which HIV researchers measure mobility.

This review aimed to describe and critically evaluate mobility measures used in HIV research in SSA over the last decade to update our understanding of current measurement approaches, add clarity to a growing body of literature, identify future research directions and guide HIV researchers seeking to measure mobility. Better mobility measures can inform HIV programmes with actionable evidence to meet the diverse and growing needs of people on the move, facilitating progress towards epidemic control in the region.

## 2 METHODS

### 2.1 Study design

This scoping review was conducted in accordance with PRISMA‐ScR guidelines to systematically search the SSA HIV literature and synthesize mobility measures [35, 36]. We focused on SSA since it contains countries with high HIV burdens (over 1% prevalence) [37]. We described and characterized mobility measures, situating them within their research contexts (HIV outcomes, populations and geographies) to identify evidence gaps and evaluate current measurement approaches.

### 2.2 Search strategy and study selection

The Population‐Concept‐Context framework was used to design the search strategy, which aimed to identify mobility measures (concept) used in HIV research (context) within SSA (population) [38]. Studies published from 1 January 2014 to 20 November 2023 were searched from three electronic databases: *PubMed*, *Embase* and *Web of Science*. Search strategies were built to include MeSH terms (PubMed), subject headings (Embase) and key words to identify HIV‐related and mobility‐related original research articles (Table S1). Text‐words and author affiliation fields were used to identify research from SSA countries. Key words previously found to be effective in identifying mobility literature within HIV research included variations of the terms “mobility,” “movement,” “migration” and “travel” [39, 40, 41]. These reflect the continuum of mobility, from overnight travel to permanent migration [42, 43, 44, 45]. These terms may not capture articles on specific forms of mobility like commuting or displacement.

Article screening was conducted in Covidence [46]. Original peer‐reviewed quantitative or mixed‐methods research articles were included if they investigated the relationship between mobility and an HIV indicator in an SSA country (Table S2). In addition, data must have been collected anytime from 2014 to 2023, inclusive, representing a decade of research as countries scaled up, implemented and sustained Treat‐All policies, making all persons with HIV eligible for treatment [47].

HIV research priorities in the Treat‐All era shifted towards identifying gaps in access and the unmet needs of underserved populations [48]. To support HIV service delivery, only articles on HIV indicators at the individual level (e.g. excluding modelled HIV prevalence rates) were included. Studies were included if the HIV outcome related to mobility was a Global AIDS Monitoring (GAM) indicator recommended by UNAIDS or fit along the prevention, care and treatment cascade [49]. These could include, but were not limited to, HIV prevalent infection, HIV testing, condom use, pre‐exposure prophylaxis (PrEP) use, antiretroviral therapy (ART) initiation, ART adherence, viral load suppression and AIDS‐related mortality. HIV‐related sexual behaviours like transactional sex were excluded.

From December 2023 to March 2024, two reviewers (AK and SW) first screened all de‐duplicated titles and abstracts from the three databases for general relevance. Then, in screening relevant full texts for inclusion, reviewers recorded the primary reason for exclusion according to the criteria listed in Table S2. Reviewers independently screened and then met to discuss and resolve any disagreements. A third reviewer (KG) served as a tie breaker if needed in final inclusion decisions. Cohen's kappa statistics described the inter‐rater reliability between the two reviewers.

### 2.3 Data extraction and analysis

An extraction form was created to chart data from each article representing study characteristics, the research context and key metrics used to measure mobility. After all coauthors agreed on the fields of interest, AK and SW extracted data from five randomly selected articles, discussed any discrepancies and further calibrated the form [50]. AK extracted data from the remaining articles.

Information on the research context included the HIV outcome(s) under study, study population and geographic context (physical geographies and local socio‐economic drivers of mobility). HIV outcomes were classified according to official indicator definitions in the 2024 GAM guidance [51]. Study populations were described in terms of their age and gender makeup, and whether they represent general or key populations (sex workers, men who have sex with men, transgender people and people who inject drugs) [1]. Other priority groups like pregnant women or specific labour groups were considered. Mobility measure information included definitions, reference periods and geographies, units of analysis, specific metrics, and data collection and analytic methods—some of which have been collected in previous reviews [3, 40].

Definitions of mobility were classified according to the International Organization for Migration (IOM) glossary and related policy documents, regardless of the label used by the study authors [52, 53, 54]. Within the umbrella term of human mobility, IOM defines migration as changing one's place of “usual residence,” being a place where one lives and spends “their daily period of rest.” Migration can be permanent (moving with the intent to stay), temporary (moving for a specific purpose and limited time with the intent to return to the origin location) or circular (repeated movements back and forth between two or more locations). Definitions of mobility that do not entail a change in usual residence were termed travel, defined as movement “between different geographic locations, for any purpose or duration.”

**Figure 1:**
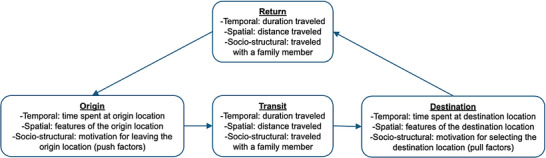
Stages and dimensions of movement used to characterize mobility metrics with examples.

Within these definitions, metrics may be used to categorize distinct mobile sub‐groups or quantify mobility on a continuous scale. As shown in Figure 1, a combination of theories was used to characterize such metrics. First, metrics were categorized according to the stage of movement described (origin, transit, destination and return). This was informed by the IOM framework for mobility and HIV which acknowledges that HIV‐related vulnerability relates to particular stages of movement, and that HIV interventions should target specific stages [55]. Second, within each stage, the temporal, spatial or socio‐structural dimension of the metric was described. This was informed by Sheller and Urry's mobilities theory which stresses the spectrum of human mobility, meaning that people move over varying spatial and temporal scales [44]. Everett Lee's theory of migration stresses the socio‐structural dimension of mobility, like push and pull factors that drive someone to leave their origin location or choose their destination location [56]. Metrics describing these dimensionalities are typically lacking in mobility health research [42, 57, 58], but are important for HIV: features like time spent away from home, distance travelled and the motivation for movement can differentially influence HIV outcomes [6, 25].

## 3 RESULTS

### 3.1 Description of included studies

**Figure 2:**
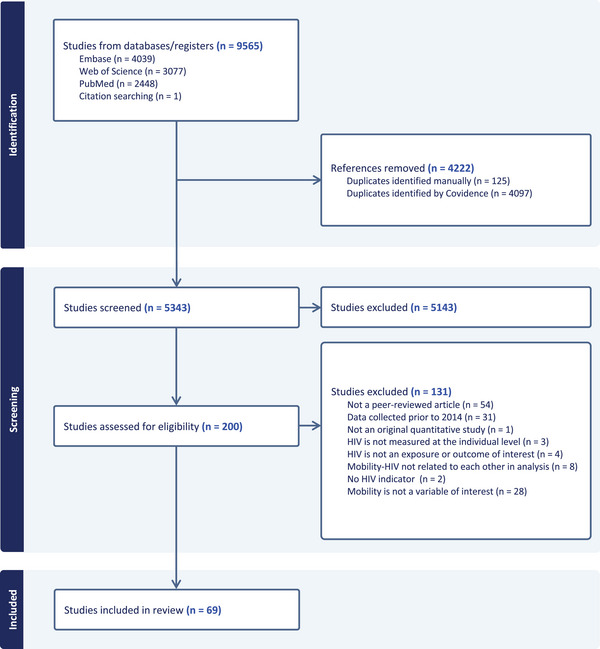
PRISMA flow chart.

Of 5343 de‐duplicated studies, 69 (1%) studies were included in the final sample (Figure 2). Agreement between reviewers was high for screening both titles/abstracts (Cohen's Kappa: 0.68) and full texts (0.75).

**Figure 3:**
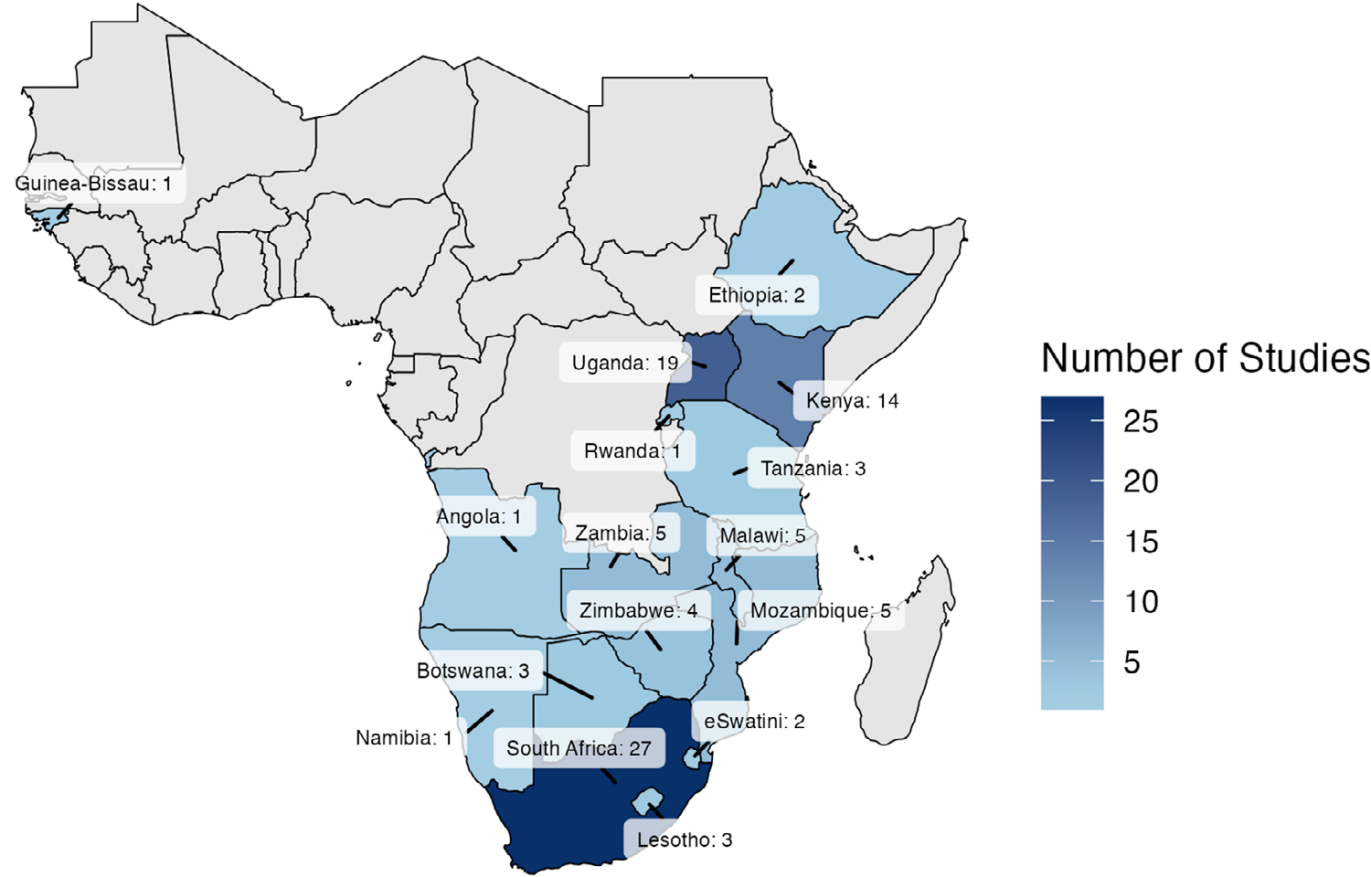
Number of studies examining the relationship between mobility and HIV in sub‐Saharan Africa, 2014–2023.

Studies represented 16 countries in SSA, with South Africa (27), Uganda (19) and Kenya (14) representing 63% of all identified studies (Figure 3). Only one study was conducted in west Africa (Guinea‐Bissau) and none in central Africa.

### 3.2 Mobility measurement

#### 3.2.1 Definitions

Across 69 studies, mobility was defined as migration and travel in 51 and 21 studies, respectively. Since four studies used both migration and travel as separate definitions of mobility, and two studies used a combination of migration and travel definitions, there were a total of 74 definitions used across the 69 studies (Table 1). Migration‐specific definitions were operationalized in one of three ways (Table S3). First, migration was described as the process of changing the place of usual residence (e.g. moving into a study area to establish a new primary residence [9]) (39/51, 76%). Second, migration was described as the length of time one has resided in their current place of residence since their last move (4/51, 8%). Third, migration was defined according to the *person* being identified as migrant, with no description of the migration process (8/51, 16%). For example, three Botswanan studies defined migrants as someone who was born elsewhere and was a “non‐citizen” [59, 60, 61].

**Table 1:** Count of mobility definitions

Definition	Count of studies
**Migration**: Movement away from/change in place of usual residence	51
Process‐specific: migration experience	39
Process‐specific: length of residence	4
Person‐specific	8
**Travel**: Movement between geographic locations	21
**Combined**: Either migration or travel	2
**Total number of studies**	69

Travel‐specific definitions usually described mobility as spending a minimum period of time (e.g. a night or month) away from the place of usual residence (*n* = 17/21, 81%). Four (19%) travel‐specific studies broadly defined travel as movement between geographies, like if participants reported “working elsewhere” [62]. No studies explicitly captured daily (non‐overnight) movement.

Since studies further restricted their definitions of a mobile group spatially or temporally (e.g. those who were away from home for at least four consecutive nights at least three times in the past 12 months) [63], mobility measures were rarely repeated across studies (Table S3).

Both migration and travel were typically defined by reference periods and geographies. Reference periods specified the time interval in which mobility experiences were captured. Periods were often the past “x” number of years/months/days before being surveyed but also included specific life stages like the time since giving birth, since “x” age, or before knowledge of HIV seroconversion. In four (6%) studies, the reference period was unclear or unknown.

Reference geographies specified the place or space to which movement was related, or the geographic “anchor” [64]. For example, a question asking, “How many nights did you spend away outside your home district?” used a subnational area as the reference geography—capturing only mobilities that occurred over certain spatial scales [65]. Other reference geographies included the “home” or primary residence, the community, the surveillance/study area or country. In six (9%) studies, the reference geography was unclear or unknown. For example, one study categorized participants as non‐migrants and internal migrants, with no explanation of which geographic border that internal migrant must have crossed in order to be identified as a “migrant” [66].

In addition to reference periods and geographies, migration and travel were also defined by the unit of analysis. Among migration measures, 43 (81%) captured individual‐level migration. Ten (14%) studies captured migration at other levels, like migration of the caregiver or mother, children within the household, sexual or marital partners and migration rates within a geographic area. Only two (10%) of the 21 travel‐specific studies captured travel at levels other than the individual [62, 63].

Data were most often collected via individual survey questionnaires (43, 62%) or household census forms available via population‐based cohort studies (20, 29%). Six (9%) studies collected mobility information via routine data sources, such as electronic medical records, programme monitoring data or workplace registries. These were limited to the person‐specific definitions of migration, such as someone being recorded as a migrant in their health record [59, 60, 61]. No studies collected mobility data via GPS‐based tools like wearable GPS devices or mobile phones, though GPS was sometimes used to geocode residences or named locations.

#### 3.2.2 Metrics

Beyond binary mobility definitions, 37 (54%) studies used a wide range of metrics to further categorize or quantify mobility (Table S4). This made it possible to detect different levels of risk between sub‐groups or over temporal and spatial scales. Metrics represented spatial, temporal and socio‐structural dimensions of movement in 17 (25%), 32 (46%) and 12 (17%) studies, respectively. Only 15 (22%) studies examined more than one dimension, with nine (13%) examining all three dimensions.

Across studies, a total of 111 specific metrics were used, representing various dimensions and stages of movement (origin, transit, destination, return) (Table 2). Of these, 46 (41%) used a temporal aspect to describe the significance of one's mobility experience, like the length of time spent at an individual destination, or the total amount of time spent away from the origin location.

**Table 2:** Count of metrics used to measure mobility by stage and dimension of movement across included studies

Stage	Spatial (37)	Temporal (46)	Socio‐structural (28)
Origin (10)	○ Origin location (1) ○ Location features (3): migration rate, linguistic area, rural/urban		○ Motivation (push factors) (3): divorce, death of a spouse, loss of employment, fleeing from violence, sent by family ○ Delivered at origin (1) ○ Frequency of remittances (1) ○ Perceived migration success of household (1)
Transit (37)	○ Distance travelled/migrated (4): distance by location, maximum distance, cumulative distance, median circuit distance ○ Borders (14): inter/intra study area, community, regional, national ○ In versus out‐migrating (2)	○ Frequency of trips/moves in a given period (12)	○ Accompaniment (3): child, husband, family member ○ Travelled with passport (1) ○ Age at time of mobility event (1)
Destination (62)	○ Destination location (3) ○ Number of unique locations (3) ○ Location features (3): migration rate, linguistic area, rural/urban ○ Type of destination community (2): fishing village, town/city, growth point, mine, farm ○ Destination is job location (1) ○ Availability of the health programme at destination (1)	○ For temporary movements, duration of stay (16): time spent away on most recent trip, cumulative time spent away, average time spent away ○ For permanent movements, length of residency/time since arrival (12) ○ Percentage of time spent away or living outside the origin location (4)	○ Motivation (pull factors) (9): work, non‐work, visiting friends/relatives, caretaking, to be cared for, to seek healthcare, to join partner, for religious ceremonies, for marriage, to start a new household, for education, for improved housing, for trading, farming, fishing, working in sex work, for opportunity, for family ○ Residence at destination (2) ○ Destination home is owned/rented (1) ○ Delivered at destination (1) ○ Attended a health facility at destination (1) ○ Worked in sex work at destination (1) ○ Used healthcare at destination (1) ○ Likelihood of accessing healthcare at destination if sick (1)
Return (2)		○ Recency of/time since last travel (2)	

*Note*: “Time” refers to the number of days, nights, weeks, months or years, depending on the study and reference period.

Spatial metrics (37/111, 33%) described more stages of movement. Like duration, distance was used to describe the significance of mobility, like in the spatial boundaries one crossed, or the number of kilometres travelled. For example, 14 studies categorized people on the move into intra‐regional, inter‐regional and inter‐national migrants/travellers. Other spatial metrics were place‐based, describing key features of the origin and destination locations, and sometimes the relation between the two. For example, travellers were categorized by whether they travelled to a region of the country with a different linguistic area from their own [67]. Another study categorized travellers by whether they had visited a fishing community versus a rural village versus a town [68].

Fewer metrics (28/111, 25%) described socio‐structural dimensions of movement. Metrics included the push and pull factors that motivated movement, whether the individual was accompanied by family on the journey or at the destination and significant activities that took place at origin or destination locations (e.g. accessing healthcare [67] or giving birth [69]). Exact motivations varied greatly across studies, but some studies grouped motivations into broader categories like work versus non‐work [25, 62], or travelling for opportunity versus after loss (e.g. divorce or job loss) versus for family [25, 63].

### 3.3 Research contexts

#### 3.3.1 HIV outcomes

**Figure 4:**
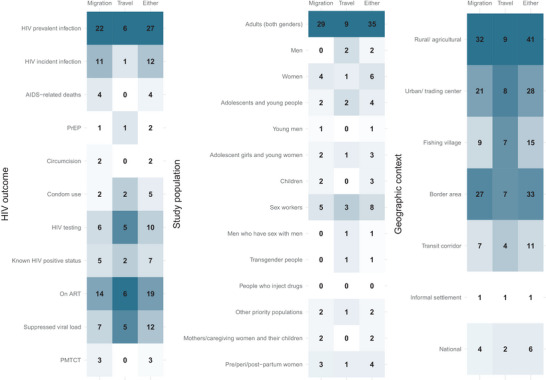
Number of studies examining mobility by definition and research contexts: HIV outcome of interest, study population and geographic context. *Note*: The two studies with combined definitions of mobility were considered to have examined “either” definition of mobility, not migration alone or travel alone. “Other priority populations” include three studies with female market traders and one study with internal migratory workers. Geographic contexts are those explicitly mentioned by the authors or in associated papers of the parent study. ART, antiretroviral therapy; PMTCT, prevention of mother‐to‐child transmission; PrEP, pre‐exposure prophylaxis.

Mobility was examined in relation to a variety of HIV indicators, even within individual studies (Figure 4 and Table S5). Many of the 69 studies related mobility to care and treatment outcomes among persons with HIV (38, 55%) or epidemiologic outcomes like HIV prevalent (27, 39%) or incident (12, 17%) infection. ART use was the most studied programmatic indicator (19, 28%), including a range of outcomes like linkage to care (1), enrolment in care (2), retention in care (8), ART initiation (3), ART coverage (9) and ART adherence (6). Compared to studies investigating care and treatment outcomes, fewer studies examined HIV testing (10, 14%), or prevention interventions (9, 13%), like PrEP (2), condom use (5) and circumcision (2).

Definitions and metrics differed based on the HIV outcome under study. For example, HIV prevalent infection was more often studied in relation to migration (22) compared to travel (6). Of the 14 studies measuring socio‐structural dimensions of mobility, only two examined these metrics against HIV testing or prevention outcomes—most studied care and treatment outcomes or HIV prevalent infection. No prevention of mother‐to‐child transmission‐focused study used any metric to quantify or categorize mobility.

#### 3.3.2 Populations

Most study populations included adults (men, women or both aged 15 and above) in the general population (43, 62%), 33 (77%) of which participated in studies on migration. Fewer of the 69 studies were among specific populations like children (3, 4%), adolescents or young people of any gender (8, 12%), pre/peri/post‐partum women (4, 6%) and key populations (10, 14%). Key populations‐focused studies mostly included female sex workers (8), with only one study enrolling men who have sex with men and transgender women [70]. Two (3%) studies were conducted among other locally prioritized populations like female market traders and migrant workers. Whether they were specifically enrolled as sex workers, female market traders, transgender women, mothers, caregivers, pregnant people or members of the general population, 25 (36%) studies focused on women and girls. Only five (7%) studies focused on men. When more than one age group or gender was under study, 25 (36%) and 24 (53%) studies stratified mobility measures by age and gender, respectively (Table S6).

Metrics differed based on the study population. For example, two of the three studies conducted among postpartum women captured whether healthcare was accessed in the origin versus destination location. Multidimensional metrics were especially lacking for some populations, like among adolescents and young people whose HIV outcomes were never related to a socio‐structural metric of mobility and only once related to a spatial metric.

#### 3.3.3 Geographies

Studies were conducted across a range of geographic contexts, including rural agricultural zones, urban centres (including both trading towns and capital cities), fishing villages, border areas, transit corridors and informal settlements. Migration was more often studied than travel in rural, urban and border areas, while migration and travel were more evenly studied in fishing villages and transit corridors. Geographic settings largely represented the disproportionate placement of studies in and around the eastern region of South Africa, and around Lake Victoria in east Africa. This includes 12 studies from the Africa Health Research Institute Health and Demographic Surveillance System in KwaZulu‐Natal, South Africa [34], seven from the Sustainable East Africa Research in Community Health (SEARCH) trial communities across Kenya and Uganda [71], and five from the Rakai Community Cohort Study (RCCS) in southern Uganda [72]. Metrics varied based on the geographic context, but variation could reflect the availability of mobility data in these specific parent studies. Nationally representative studies using Demographic & Health Surveys, for example, only used a binary definition of migration or travel.

## 4 DISCUSSION

Mobility measures varied widely and were inconsistently studied across HIV outcomes, populations and geographies. HIV researchers employed diverse definitions and metrics—often capturing just one dimension of mobility at a time—hindering efforts to systematically evaluate the relationship between different forms of mobility and HIV outcomes. We found an overarching need to fill evidence gaps, standardize reporting and design comprehensive mobility measures that are relevant to the local setting and situated within an HIV transmission framework.

### 4.1 Actionable evidence for HIV epidemic control

Mobility measures were rarely used in more than one study even within the same country or data source. Differences in reference periods and geographies, or restricted definitions of mobile sub‐populations, led to non‐comparability. Standardizing mobility measures could help synthesize evidence on mobility and HIV to inform HIV epidemic control.

Studies often lacked standardization in mobility metrics because researchers tailored mobility metrics to reflect the known forms of mobility in their setting or population. Such contextualized measures have two main benefits. First, they allow programmes to identify blind spots in the HIV response. For example, in rural Mozambique, where men migrate for work while their female spouses stay behind, Agadjanian and colleagues used socio‐structural metrics like perceived migration success and frequency of remittances to understand households' collective migration experience [73]. These metrics helped identify households not benefiting from migration and women's associated health risks. Second, contextualized measures can be more reliable. For example, circuit distance (the median of all possible paths) can more accurately capture the mobility of sex workers, who tend to move between destinations to appear “new” to potential clients [62]. Since simply summing distances between home and each destination would have misestimated mobility, this tailored measure allows for a more accurate assessment of mobility and HIV risk.

**Figure 5:**
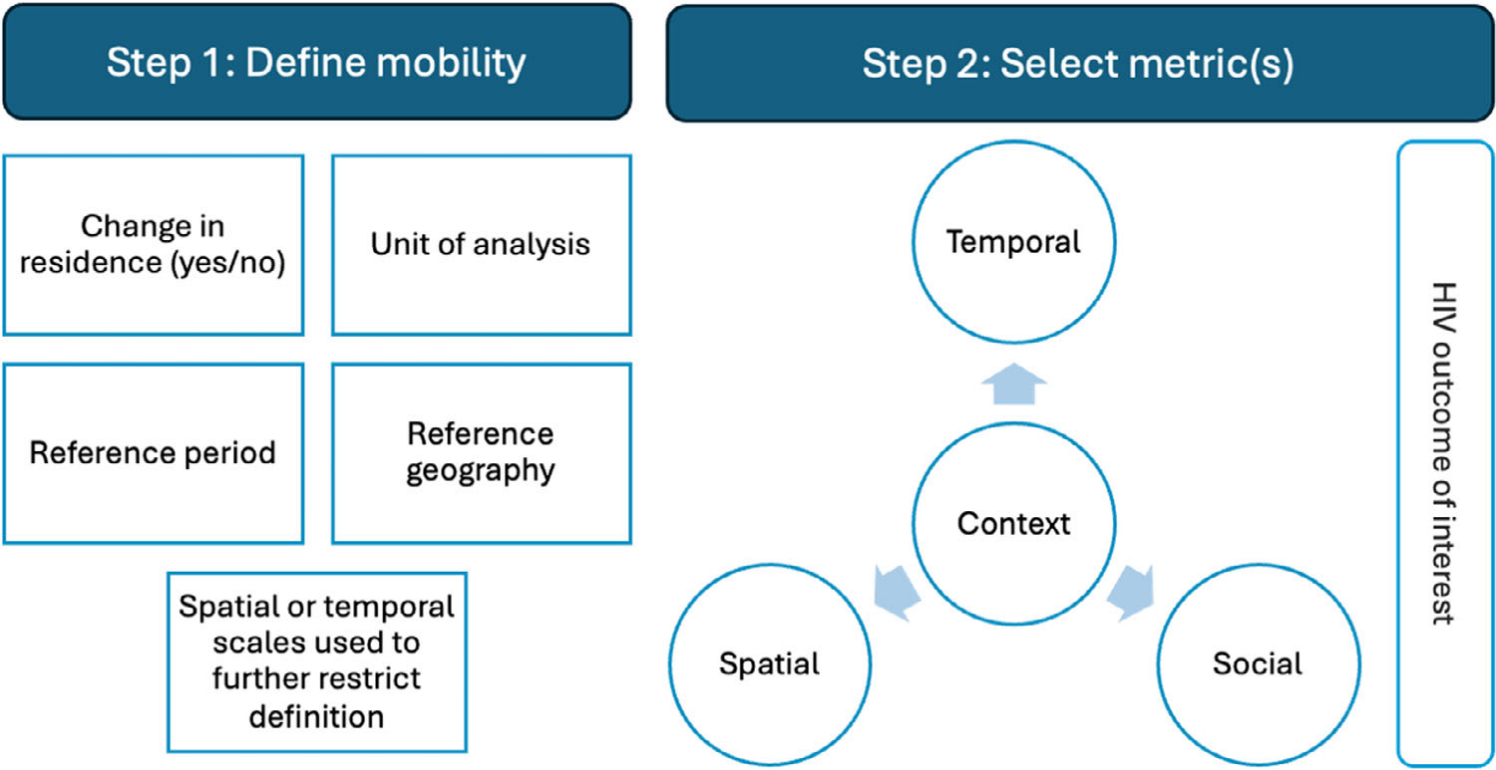
Framework for designing mobility measures in HIV research.

Together, standard definitions and contextualized metrics of mobility can generate actionable evidence for HIV epidemic control—aiding both evidence synthesis and the production of locally relevant knowledge for programmatic change. Producing such detailed measures may be challenging with limited budgets. Thus, we propose a framework for designing mobility measures (Figure 5). First, mobility can be defined according to IOM definitions of migration or overnight travel [52, 53, 54]. Every mobility definition should include the unit of analysis, reference period, reference geography and any spatial or temporal scales used to identify the mobile population of interest, like minimum duration of travel. Consistently reporting these components in scientific research can aid the interpretation of results across studies.

Second, within these broad categories, multidimensional metrics can more comprehensively describe local mobility patterns to relate different forms of mobility to HIV outcomes. Yet, many studies examined one dimension of mobility at a time, and few included socio‐structural metrics like the motivation for movement. The research context, including structural forces that drive human movement, can guide which metrics to capture (Figure 1). For example, around Uganda's Lake Albert, metrics included the number of trips between fish landing sites [74], while in a rural southern Mozambican district, metrics included the length of time spent living outside the country [75], as men move around the region for mining. It may not be immediately clear what is locally relevant; qualitative research and participatory methods like involving stakeholders and community members in study design can provide invaluable insights, revealing potentially important mobility patterns worth measuring [76, 77].

To further guide metric selection, researchers should situate mobility within an HIV transmission framework, prioritizing metrics most relevant to reducing incidence or HIV‐related morbidity and mortality. First, causal diagrams and hypothesized mechanisms of action can help identify which mobility dimensions relate to specific HIV outcomes [78]. For example, spatial metrics were often related to HIV acquisition and condom use, reflecting social control theory in which greater distance from home increases sexual behaviours that impact HIV transmission [79, 80, 81]. Second, considering HIV transmission dynamics helps to design relevant measures. For example, Dobra and colleagues defined communities based on geographically varying incidence rates, comparing HIV epidemic typologies between origin and destination communities [82]. Mobility measures became more useful for epidemic control: if most individuals move from high‐ to low‐incidence communities, then HIV prevention efforts should focus on not just where the highest numbers of people with HIV live but where they recently lived, especially if those communities have significant out‐migration [83].

Improved mobility measurement can better inform HIV programmes working to end the epidemic. At an individual level, mobility research can identify populations in need of tailored interventions. This could include long‐acting PrEP for people who travel for long periods or connecting postpartum women and their infants to care as they migrate [84, 85]. At a population level, research can identify mobile populations with a disproportionate impact on HIV transmission. Mobility measures could also directly inform implementation strategies. One review of disease transmission interventions for mobile populations highlighted the approach of offering services at multiple sites and times to account for where and when people move around [86]. This can increase service coverage but requires detailed mobility metrics on the locations and timing of people's movement. Further, to secure such detailed metrics, innovative data collection methods must identify, follow and collect consistent data on mobile populations who are often missing or lost to follow up in health research. This may include leveraging ecological momentary assessments to collect data [87] or enrolling participants via respondent‐driven or starfish sampling methods [88, 89].

### 4.2 Evidence gaps

Even as research highlights the HIV risks experienced by many people on the move, evidence gaps remain for prevention programmes, across west and central Africa, among key populations, and by gender. Mobility and HIV research in SSA disproportionately focuses on care and treatment for people with HIV compared to HIV prevention. There is limited knowledge about HIV acquisition risk among travellers or PrEP use for any mobile group. This evidence is crucial: as countries reach 95‐95‐95 targets, primary prevention is likely to become a focus for reducing new HIV acquisitions. This is especially true as tools like long‐acting injectable PrEP could provide better options for people on the move [85].

Longitudinal cohorts and population‐based surveys in eastern and southern Africa have greatly enhanced our understanding of mobility and HIV in SSA, but data are severely lacking in west and central Africa, where countries lag in meeting 95‐95‐95 targets and HIV incidence declines more slowly [1]. Studies in South Africa and around Lake Victoria capture many forms of mobility, like circular labour migration, short‐term travel for the fishing industry and trade‐related travel. However, relying solely on these settings assumes that local cultural contexts can be extrapolated elsewhere. Population mobility may differ in its spatial, temporal and socio‐structural dimensions, and in its relationship to HIV [16]. For instance, Burkina Faso has one of the highest out‐migration rates in West Africa yet is one of the most rural countries in the region [90]. To adapt HIV programmes to increasing population mobility across SSA, diversifying the evidence base is crucial.

As the epidemic declines, HIV programmes need data on mobile key and other priority populations. Sex workers frequently travel between communities, sexual and gender minorities may move to escape violence and people who inject drugs often experience housing instability [3, 91, 92]. Once mobile, the already disproportionate HIV risks experienced by these groups can worsen [3]. Among sex workers in SSA, research on mobility and HIV is growing but has focused mainly on condom use or HIV prevalent infection. Given that up to 62% of sex worker populations in SSA are living with HIV, research on mobility and HIV treatment can aid programmes [1]. The single study included in this review among men who have sex with men and transgender women in Nairobi underscores the urgent need for mobility research among sexual and gender minorities in SSA [70]. With intensifying anti‐homosexuality and transphobic law enforcement and related community violence, many are on the move, yet key population programmes have no data on which to act. Finally, ending the epidemic may require identifying new key populations, and mobility research can help. For example, over one‐third of female market traders in Kenya had travelled overnight in the past month, and one‐quarter were living with HIV [93]. Effective implementation strategies for these populations are essential.

Finally, relationships between mobility and HIV were rarely stratified by gender. Mobility is a highly gendered process that varies over the life course [58, 94] and its effects on HIV may differ among men and women [58]. Women may need to travel for longer [6] or shorter [95] periods of time to experience the same increases in HIV incidence as men. Furthermore, mobile men experience low rates of uptake and retention in care and treatment programmes [26, 96], yet few studies enrolled men specifically to investigate these relationships. However, it is possible that such studies were not detected by our search if they included specific male mobile groups like truck drivers and seasonal miners without mentioning mobility explicitly.

In an update to our review to April 2025, we found six studies [96, 97, 98, 99, 100, 101]; four were concerned with migration [96, 97, 98, 99] and two with travel [100, 101]. Four were conducted in Uganda [98, 99, 100, 101]. Two focused on non‐migrants exposed to household migration [98, 99]. Only one considered spatial distance [100], while only three included social‐structural dimensions [96, 100, 101], highlighting the need for the proposed framework in Figure 5. While two studies focused or reported specifically on men [96, 101], evidence gaps remain for West and Central Africa and key populations.

### 4.3 Strengths and limitations

The search strategy may have had limited sensitivity for detecting specific forms of mobility, namely: daily commuting, forced displacement and nomadic pastoralism. Growing bodies of literature in SSA on internally displaced populations and refugees [91, 102, 103] and nomadic pastoralists [104, 105] show that these populations experience many HIV‐related risks. Similarly, daily commuting may be highly relevant to HIV in SSA: some programmes prioritize occupational groups like motorbike and minibus drivers for HIV services [106, 107]. In Johannesburg, rural‐urban commuters may be less likely to use HIV testing and counselling services [108, 109]. Relationships between mobility and HIV in these populations deserve more in‐depth analyses. Collecting GPS‐based activity space data can be a promising method for describing finer‐scale mobility, even in low‐resource settings [87]. Yet, we found no studies using GPS‐based mobility measures.

While mobility has been implicated in the spread of HIV in SSA since the start of the epidemic [110], this is the first study to systematically review mobility measures. It lays a critical foundation for future research, enabling HIV programmers to reconcile evidence on various forms of mobility in their population, study implementers to design comprehensive measures and HIV researchers to evaluate the comparability of mobility measures for meta‐analysis. By employing theories from geography, demography and epidemiology, this review clarifies the complexity of mobility and its relationship with HIV [44]. An HIV transmission framework helped to evaluate measures across a range of HIV outcomes, while other reviews have previously focused singularly on HIV acquisition [40] or HIV treatment [33]. Finally, we rigorously applied systematic review methods to achieve high inter‐rater reliability in screening articles.

## 5 CONCLUSIONS

People on the move are a vast and diverse group with nuanced HIV‐related needs, yet they are often labelled as one “priority population” [111]. To unpack the relationship between mobility and HIV, researchers must embrace the complexity of human movement and design multidimensional and locally relevant measures, with standard reporting of the mobility definition. Further, by situating mobility within the broader goals of HIV epidemic control and an equitable HIV response, researchers can create actionable evidence for more effective HIV programming. This may aid efforts to end HIV as a public health threat by 2030 while also securing migrants' rights to healthcare [24].

Improved measurement of mobility has the potential to change the course of the epidemic. Robust research on HIV programmatic gaps experienced by people on the move may reduce inequalities in service access and in the risk of infection—not just among mobile people but also in their networks. As SSA faces record rates of human mobility driven by social, economic and environmental forces, the HIV response must adapt to the realities of people on the move, relying on detailed data to guide its efforts.

## COMPETING INTERESTS

The authors have no conflicts of interest to disclose.

## AUTHORS' CONTRIBUTIONS

AK, BM and MKG conceived of the research question. AK developed the theory and designed the study methods with input from BM, MKG, DTD, FN and QAK. AK conducted the search. AK and SW screened articles for inclusion and extracted data with input from BM, MKG and DTD. AK analysed the data and wrote the manuscript in consultation with all other authors. All authors read and approved the final manuscript.

## FUNDING

Research reported in this publication was supported by the National Institute of Mental Health (F31MH134699, PI: Khalifa) and the National Institute of Allergy and Infectious Diseases of the National Institutes of Health (T32AI114398). Dustin T. Duncan's time was supported in part by grants from the National Institute on Minority Health and Health Disparities (R01MD013554, R01MD013554‐S1), the National Institute on Mental Health (R01MH129198, P30MH043520), the National Institute on Drug Abuse (R01DA054553, P30DA011041), the National Heart, Lung and Blood Institute (R01HL160325) and the National Institute on Allergy and Infectious Diseases (UG3AI169658).

## DISCLAIMER

The findings and conclusions in this paper are those of the authors and do not necessarily represent the official position of the funding agencies.

## Supporting information




**Table S1**. Search terms by database.
**Table S2**. Inclusion and exclusion criteria.
**Table S3**. Study‐specific mobility definitions and methods.
**Table S4**. Study‐specific mobility metrics by dimension.
**Table S5**. Study‐specific research contexts.
**Table S6**. Study‐specific age and gender stratifications.

## Data Availability

The data that support the findings of this study are available in the Supplementary Material of this article.
