# Supplementary material for: Measuring mobility in HIV research in sub‐Saharan Africa: a scoping review
Source: J Int AIDS Soc. 2025 Jun 5;28(6):e26508. doi: 10.1002/jia2.26508 (PMC12141761; doi:10.1002/jia2.26508)
Supplement: Supplementary file 1 — Table S1. Search terms by database. Table S2. Inclusion and exclusion criteria. Table S3. Study‐specific mobility definitions and methods. Table S4. Study‐specific mobility metrics by dimension. Table S5. Study‐specific research contexts. Table S6. Study‐specific age and gender stratifications. [file JIA2-28-e26508-s001.docx]

Supplement

Table of Contents

[Table S1. Search terms by database 2](#_Toc184728262)

[Table S2. Inclusion and exclusion criteria 3](#_Toc184728263)

[Table S3. Study-specific mobility definitions and methods 4](#_Toc184728264)

[Table S4. Study-specific mobility metrics by dimension 12](#_Toc184728265)

[Table S5. Study-specific research contexts 21](#_Toc184728266)

[Table S6. Study-specific age and gender stratifications 30](#_Toc184728267)

## Table S1. Search terms by database

| **Database** | **Search** |
| --- | --- |
| PubMed | *("HIV"[Title/Abstract] OR "AIDS"[Title/Abstract] OR "hiv infections/epidemiology"[MeSH Terms] OR "hiv infections/diagnosis"[MeSH Terms] OR "hiv infections/mortality"[MeSH Terms] OR "hiv infections/prevention and control"[MeSH Terms] OR "hiv infections/transmission"[MeSH Terms])* |
|  | *AND ("mobil*"[Title/Abstract] OR "migra*"[Title/Abstract] OR "move*"[Title/Abstract] OR "travel*"[Title/Abstract])* |
|  | *AND 2014/01/01:3000/12/31[Date - Publication]* |
|  | *AND ("Africa South of the Sahara"[MeSH Terms] OR "africa*"[tw] OR "Malawi"[tw] OR "Liberia"[tw] OR "Nigeria"[tw] OR "South Africa"[tw] OR "Ghana"[tw] OR "Tanzania"[tw] OR "Zanzibar"[tw] OR "Kenya"[tw] OR "Rwanda"[tw] OR "Botswana"[tw] OR "Senegal"[tw] OR "Angola"[tw] OR "Uganda"[tw] OR "Mali"[tw] OR "Sierra Leone"[tw] OR "Ivory Coast"[tw] OR "Ethiopia"[tw] OR "Lesotho"[tw] OR "Zambia"[tw] OR "Zimbabwe"[tw] OR "Namibia"[tw] OR "Mauritius"[tw] OR "Mozambique"[tw] OR "Niger"[tw] OR "Seychelles"[tw] OR "Burkina Faso"[tw] OR "Burundi"[tw] OR "Cape Verde"[tw] OR "Cabo Verde"[tw] OR "Cameroon"[tw] OR "Cameroun"[tw] OR "Central African Republic"[tw] OR "Chad"[tw] OR "Comoros"[tw] OR "Democratic Republic of Congo"[tw] OR "DR Congo"[tw] OR "Djibouti"[tw] OR "Cote d'Ivoire"[tw] OR "Ivory Coast"[tw] OR "Congo"[tw] OR "Equatorial Guinea"[tw] OR "Guinea"[tw] OR "Eritrea"[tw] OR "Gabon"[tw] OR "Guinea-Bissau"[tw] OR "Madagascar"[tw] OR "Congo Republic"[tw] OR "Sao Tome and Principe"[tw] OR "Sao Tome"[tw] OR "Sao Tome and Principe"[tw] OR "Swaziland"[tw] OR "Eswatini"[tw] OR "Togo"[tw] OR "Benin"[tw] OR "Gambia"[tw] OR "Mauritania"[tw] OR "Malawi"[Affiliation] OR "Liberia"[Affiliation] OR "Nigeria"[Affiliation] OR "South Africa"[Affiliation] OR "Ghana"[Affiliation] OR "Tanzania"[Affiliation] OR "Zanzibar"[Affiliation] OR "Kenya"[Affiliation] OR "Rwanda"[Affiliation] OR "Botswana"[Affiliation] OR "Senegal"[Affiliation] OR "Angola"[Affiliation] OR "Uganda"[Affiliation] OR "Mali"[Affiliation] OR "Sierra Leone"[Affiliation] OR "Ivory Coast"[Affiliation] OR "Ethiopia"[Affiliation] OR "Lesotho"[Affiliation] OR "Zambia"[Affiliation] OR "Zimbabwe"[Affiliation] OR "Namibia"[Affiliation] OR "Mauritius"[Affiliation] OR "Mozambique"[Affiliation] OR "Niger"[Affiliation] OR "Seychelles"[Affiliation] OR "Burkina Faso"[Affiliation] OR "Burundi"[Affiliation] OR "Cape Verde"[Affiliation] OR "Cabo Verde"[Affiliation] OR "Cameroon"[Affiliation] OR "Cameroun"[Affiliation] OR "Central African Republic"[Affiliation] OR "Chad"[Affiliation] OR "Comoros"[Affiliation] OR "Democratic Republic of Congo"[Affiliation] OR "DR Congo"[Affiliation] OR "Djibouti"[Affiliation] OR "Cote d'Ivoire"[Affiliation] OR "Ivory Coast"[Affiliation] OR "Congo"[Affiliation] OR "Equatorial Guinea"[Affiliation] OR "Guinea"[Affiliation] OR "Eritrea"[Affiliation] OR "Gabon"[Affiliation] OR "Guinea-Bissau"[Affiliation] OR "Madagascar"[Affiliation] OR "Congo Republic"[Affiliation] OR "Sao Tome and Principe"[Affiliation] OR "Sao Tome"[Affiliation] OR "Sao Tome and Principe"[Affiliation] OR "Swaziland"[Affiliation] OR "Eswatini"[Affiliation] OR "Togo"[Affiliation] OR "Benin"[Affiliation] OR "Gambia"[Affiliation] OR "Mauritania"[Affiliation])* |
| Embase | *(“HIV”:ti,ab,kw OR “AIDS”:ti,ab,kw OR “Human immunodeficiency virus infection”/exp)* |
|  | *AND (mobil*:ti,ab,kw OR migra*:ti,ab,kw OR travel*:ti,ab,kw OR move*:ti,ab,kw)* |
|  | *AND [2014-2023]/py* |
|  | *AND (“africa”/exp OR “africa*”:ti,ab,kw,ca,ff OR “Malawi”:ti,ab,kw,ca,ff OR “Liberia”:ti,ab,kw,ca,ff OR “Nigeria”:ti,ab,kw,ca,ff OR “South Africa”:ti,ab,kw,ca,ff OR “Ghana”:ti,ab,kw,ca,ff OR “Tanzania”:ti,ab,kw,ca,ff OR “Zanzibar”:ti,ab,kw,ca,ff OR “Kenya”:ti,ab,kw,ca,ff OR “Rwanda”:ti,ab,kw,ca,ff OR “Botswana”:ti,ab,kw,ca,ff OR “Senegal”:ti,ab,kw,ca,ff OR “Angola”:ti,ab,kw,ca,ff OR “Uganda”:ti,ab,kw,ca,ff OR “Mali”:ti,ab,kw,ca,ff OR “Sierra Leone”:ti,ab,kw,ca,ff OR “Ivory Coast”:ti,ab,kw,ca,ff OR “Ethiopia”:ti,ab,kw,ca,ff OR “Lesotho”:ti,ab,kw,ca,ff OR “Zambia”:ti,ab,kw,ca,ff OR “Zimbabwe”:ti,ab,kw,ca,ff OR “Namibia”:ti,ab,kw,ca,ff OR “Mauritius”:ti,ab,kw,ca,ff OR “Mozambique”:ti,ab,kw,ca,ff OR “Niger”:ti,ab,kw,ca,ff OR “Seychelles”:ti,ab,kw,ca,ff OR “Burkina Faso”:ti,ab,kw,ca,ff OR “Burundi”:ti,ab,kw,ca,ff OR “Cape Verde”:ti,ab,kw,ca,ff OR “Cabo Verde”:ti,ab,kw,ca,ff OR “Cameroon”:ti,ab,kw,ca,ff OR “Cameroun”:ti,ab,kw,ca,ff OR “Central African Republic”:ti,ab,kw,ca,ff OR “Chad”:ti,ab,kw,ca,ff OR “Comoros”:ti,ab,kw,ca,ff OR “Democratic Republic of Congo”:ti,ab,kw,ca,ff OR “DR Congo”:ti,ab,kw,ca,ff OR “Djibouti”:ti,ab,kw,ca,ff OR “Ivoire”:ti,ab,kw,ca,ff OR “Ivory Coast”:ti,ab,kw,ca,ff OR “Congo”:ti,ab,kw,ca,ff OR “Equatorial Guinea”:ti,ab,kw,ca,ff OR “Guinea”:ti,ab,kw,ca,ff OR “Eritrea”:ti,ab,kw,ca,ff OR “Gabon”:ti,ab,kw,ca,ff OR “Guinea-Bissau”:ti,ab,kw,ca,ff OR “Madagascar”:ti,ab,kw,ca,ff OR “Congo Republic”:ti,ab,kw,ca,ff OR “Sao Tome and Principe”:ti,ab,kw,ca,ff OR “Sao Tome”:ti,ab,kw,ca,ff OR “Sao Tome and Principe”:ti,ab,kw,ca,ff OR “Swaziland”:ti,ab,kw,ca,ff OR “Eswatini”:ti,ab,kw,ca,ff OR “Togo”:ti,ab,kw,ca,ff OR “Benin”:ti,ab,kw,ca,ff OR “Gambia”:ti,ab,kw,ca,ff OR “Mauritania”:ti,ab,kw,ca,ff )* |
| Web of Science | *TS=("HIV" OR "AIDS")* |
|  | *AND TS=(mobil* OR migra* OR travel* OR move*)* |
|  | *AND PY==("2023" OR "2022" OR "2021" OR "2020" OR "2019" OR "2018" OR "2017" OR "2016" OR "2015" OR "2014")* |
|  | *AND (CU==(“africa*” OR “Malawi” OR “Liberia” OR “Nigeria” OR “South Africa” OR “Ghana” OR “Tanzania” OR “Zanzibar” OR “Kenya” OR “Rwanda” OR “Botswana” OR “Senegal” OR “Angola” OR “Uganda” OR “Mali” OR “Sierra Leone” OR “Ivory Coast” OR “Ethiopia” OR “Lesotho” OR “Zambia” OR “Zimbabwe” OR “Namibia” OR “Mauritius” OR “Mozambique” OR “Niger” OR “Seychelles” OR “Burkina Faso” OR “Burundi” OR “Cape Verde” OR “Cabo Verde” OR “Cameroon” OR “Cameroun” OR “Central African Republic” OR “Chad” OR “Comoros” OR “Democratic Republic of Congo” OR “DR Congo” OR “Djibouti” OR “Ivoire” OR “Ivory Coast” OR “Congo” OR “Equatorial Guinea” OR “Guinea” OR “Eritrea” OR “Gabon” OR “Guinea-Bissau” OR “Madagascar” OR “Congo Republic” OR “Sao Tome and Principe” OR “Sao Tome” OR “Sao Tome and Principe” OR “Swaziland” OR “Eswatini” OR “Togo” OR “Benin” OR “Gambia” OR “Mauritania”)* |
|  | *OR TS=( “africa*” OR “Malawi” OR “Liberia” OR “Nigeria” OR “South Africa” OR “Ghana” OR “Tanzania” OR “Zanzibar” OR “Kenya” OR “Rwanda” OR “Botswana” OR “Senegal” OR “Angola” OR “Uganda” OR “Mali” OR “Sierra Leone” OR “Ivory Coast” OR “Ethiopia” OR “Lesotho” OR “Zambia” OR “Zimbabwe” OR “Namibia” OR “Mauritius” OR “Mozambique” OR “Niger” OR “Seychelles” OR “Burkina Faso” OR “Burundi” OR “Cape Verde” OR “Cabo Verde” OR “Cameroon” OR “Cameroun” OR “Central African Republic” OR “Chad” OR “Comoros” OR “Democratic Republic of Congo” OR “DR Congo” OR “Djibouti” OR “Ivoire” OR “Ivory Coast” OR “Congo” OR “Equatorial Guinea” OR “Guinea” OR “Eritrea” OR “Gabon” OR “Guinea-Bissau” OR “Madagascar” OR “Congo Republic” OR “Sao Tome and Principe” OR “Sao Tome” OR “Sao Tome and Principe” OR “Swaziland” OR “Eswatini” OR “Togo” OR “Benin” OR “Gambia” OR “Mauritania”))* |

## Table S2. Inclusion and exclusion criteria

| **Inclusion criteria** | - Study conducted in a sub-Saharan African country - Original research article - Published in a peer-reviewed journal - At least some data collected between 2014 and 2023, inclusive - Mobility is related to HIV in analysis - Mobility is a variable (covariate, predictor, exposure, or outcome) of interest - HIV is an exposure or outcome of interest - HIV indicator is at an individual level |
| --- | --- |
| **Exclusion criteria** | - Study conducted outside of sub-Saharan Africa - Systematic or scoping review, meta-analysis - Purely qualitative - Not published in a peer-reviewed journal - All data collected prior to 2014 - Mobility was not related to HIV in analysis - Studies focused on *mobile* health interventions, the functional *mobility* of elderly people living with HIV, or the *mobility* of HIV-infected cells - Mobility not measured - HIV not an exposure or outcome of interest - HIV indicator is not at the individual level |

## Table S3. Study-specific mobility definitions and methods

| **Reference** | **Title** | **Definition** | **Operational definition** | **Detailed definition** | **Reference period** | **Reference geography** | **Unit of analysis** | **Data collection method** | **Privacy/ security measures** |
| --- | --- | --- | --- | --- | --- | --- | --- | --- | --- |
| Nhampossa et al., 2021 | The impact of the caregiver mobility on child HIV care in the Manhica District, Southern Mozambique: A clinical based study | Multiple | Migration + travel | "Home-absenteeism" for over four consecutive nights at least three times in the past 12 months, or relocation (address change) | Past 12 months | Usual residence | Caregiver | Survey questionnaire | None mentioned |
| Low et al., 2021 | Migration in Namibia and its association with HIV acquisition and treatment outcomes | Migration | Process-specific: Migration experience | 1) "Significant migrant": ever lived outside Namibia or in another region inside Namibia, or lived away from home continuously for more than 1 month in the past 3 years 2) "In-migrant": "coming" from another community during the survey 2a) Recent cross-community in-migrants:  2b) Longer cross-community in-migrants: | Ever | Combination: Community if ever in-migrated, Subnational region if ever out-migrated, Usual residence if every lived away from home for more than one month | Individual | Survey questionnaire | None mentioned |
| Bahemuka et al., 2023 | Factors Associated with Short and Long Term Mobility and HIV Risk of Women Living in Fishing Communities Around Lake Victoria in Kenya, Tanzania, and Uganda: A Cross Sectional Survey | Travel | Minimum period of time away | Any overnight trip outside the village | Past 4 months | Community | Individual | Survey questionnaire | None mentioned |
| Goodman et al., 2016 | Child-street migration among HIV-affected families in Kenya: a mediation analysis from cross-sectional data. | Migration | Process-specific: Migration experience | At least one child left home to live on the street | Past 6 months | Usual residence | Household | Survey questionnaire | None mentioned |
| Odayar et al., 2023 | Mobility during the post-partum period and viraemia in women living with HIV in South Africa | Travel | Travel | At least one night was spent outside Cape Town since the last visit | 24 months since delivery | Subnational region | Individual | Survey questionnaire | None mentioned |
| Nyabuti et al., 2021 | Characteristics of HIV seroconverters in the setting of universal test and treat: Results from the SEARCH trial in rural Uganda and Kenya | Migration | Process-specific: Migration experience | Mobile: Lived away from the community for a month or more over the past 12 months | Past 12 months | Community | Individual | Survey questionnaire | None mentioned |
| Shaw et al., 2023 | Geographical Associations of HIV Prevalence in Female Sex Workers From Nairobi, Kenya (2014-2017) | Migration | Person-specific | Born in a high-prevalence county (Homa Bay, Kisumu, Migori, Siaya) that is not Nairobi | Lifetime | Subnational region | Individual | EMR/routine health record/program data | None mentioned |
| Marukutira et al., 2019-A | Clinical outcomes of a cohort of migrants and citizens living with human immunodeficiency virus in Botswana: Implications for Joint United Nation Program on HIV and AIDS 90-90-90 targets | Migration | Person-specific | Born outside of Botswana and without Botswana citizenship | Momentary | Country | Individual | EMR/routine health record/program data | None mentioned |
| Murnane et al., 2022 | Distinct forms of migration and mobility are differentially associated with HIV treatment adherence | Migration | Process-specific: Migration experience | Change in residence | Past 24 months | Usual residence | Individual | Survey questionnaire | None mentioned |
| Dobra et al., 2017 | Space-time migration patterns and risk of HIV acquisition in rural South Africa | Migration | Process-specific: Migration experience | Change in residence | Since 2004 or when person entered the cohort | Usual residence | Individual | Household census | None mentioned |
| Dobra et al., 2019 | A method for statistical analysis of repeated residential movements to link human mobility and HIV acquisition | Migration | Process-specific: Migration experience | Change in residence | Since 2004 or when person entered the cohort | Usual residence | Individual | Household census | None mentioned |
| Kiyingi et al., 2023 | Predictors of mobility among women engaged in commercial sex work in Uganda using generalized estimating equations model | Migration | Process-specific: Migration experience | Change in residence between survey time points | Past 12 months | Usual residence | Individual | Survey questionnaire | None mentioned |
| Dorward et al., 2017 | Factors associated with poor linkage to HIV care in South Africa: Secondary analysis of data from the Thol'impilo trial | Migration | Person-specific | Country of origin is not south Africa | Lifetime | Country | Individual | Survey questionnaire | None mentioned |
| Beres et al., 2021 | Patterns and Predictors of Incident Return to HIV Care Among Traced, Disengaged Patients in Zambia: Analysis of a Prospective Cohort | Travel | Minimum period of time away | Did not spend >1 month away from usual residence in the past year | Past 12 months | Usual residence | Individual | Survey questionnaire | None mentioned |
| Camlin et al., 2017 | High mobility and HIV prevalence among female market traders in East Africa in 2014 | Migration | Process-specific: Length of residence | Duration of current residency | Momentary | Usual residence | Individual | Survey questionnaire | None mentioned |
| Davey et al., 2020 | Exploring the Association Between Mobility and Access to HIV Services Among Female Sex Workers in Zimbabwe | Travel | Minimum period of time away | Either 1) took a trip in the past 12 months, 2) spent at least a day away in the past 12 months, or 3) traveled at least 1 km in the past 12 months | Past 12 months | Study area | Individual | Survey questionnaire including travel histories | None mentioned |
| Lee et al., 2023 | Condom, modern contraceptive, and dual method use are associated with HIV status and relationship concurrency in a context of high mobility: A cross-sectional study of women of reproductive age in rural Kenya and Uganda, 2016 | Multiple | Migration + travel | Either overnight travel (spending any number nights away from primary residence without an intention to change residence) or migration (relocated across district/country/country boundary to establish a new permanent residence) in the past 6 months | Past 6 months if traveled, past 24 months if migrated | Combination: Subnational region if migrated, Usual residence if traveled | Individual | Survey questionnaire including migration histories | None mentioned |
| Mthiyane et al., 2022 | The association of exposure to DREAMS on sexually acquiring or transmitting HIV amongst adolescent girls and young women living in rural South Africa | Migration | Process-specific: Migration experience | Ever moved outside or within the study area since age 13 | Since age 13 | Usual residence | Individual | Unknown | None mentioned |
| Senteza et al., 2023 | Virological non-suppression among adult males attending HIV care services in the fishing communities in Bulisa district, Uganda | Travel | Movement between geographies | Frequency of movement between fish landing sites: more than twice per year, 1-2 times per year | Past 12 months | Workplace | Individual | Survey questionnaire | None mentioned |
| Janse Van Rensburg et al., 2021 | Healthcare without borders: A cross-sectional study of immigrant and nonimmigrant children admitted to a large public sector hospital in the Gauteng Province of South Africa | Migration | Person-specific | Immigrant | Lifetime | Country | Individual | Survey questionnaire | Avoided using administrative (including medical) records to discern legal immigrant status in case of misuse of information. Instead, relied on self-reported immigrant status in private oral interviews. |
| Grabowski et al., 2021 | Prevalence and Predictors of Persistent Human Immunodeficiency Virus Viremia and Viral Rebound after Universal Test and Treat: A Population-Based Study | Migration | Process-specific: Migration experience | In-migrant: anyone who had moved into an RCCS community | Since last survey round | Community | Individual | Household census | None mentioned |
| Billioux et al., 2017 | Human immunodeficiency virus care cascade among sub-populations in Rakai, Uganda: an observational study. | Migration | Process-specific: Migration experience | In-migrant: Moved from another community since last census | Since last survey round | Community | Individual | Household census | None mentioned |
| Plymoth et al., 2020 | Socio-economic condition and lack of virological suppression among adults and adolescents receiving antiretroviral therapy in Ethiopia | Travel | Movement between geographies | Job location is outside the residential district 1) single location outside district 2) multiple locations outside district | Momentary | Subnational region | Individual | Survey questionnaire | None mentioned |
| Correa-Agudelo et al., 2021 | Associated health and social determinants of mobile populations across HIV epidemic gradients in Southern Africa. | Migration | Process-specific: Length of residence | Lived in current residence for less than a year at the time of survey | Momentary | Usual residence | Individual | Survey questionnaire | None mentioned |
| Psaki et al., 2022 | What are we learning about HIV testing in informal settlements in KwaZulu-Natal, South Africa? Results from a randomized controlled trial | Migration | Process-specific: Length of residence | Lived in the community for 0-12 months, 13-24 months | Momentary | Community | Individual | Survey questionnaire | None mentioned |
| Low et al., 2019-A | Association between severe drought and HIV prevention and care behaviors in Lesotho: A population-based survey 2016-2017 | Migration | Process-specific: Migration experience | Lived outside of Lesotho in the past 12 months | Past 12 months | Country | Individual | Survey questionnaire | None mentioned |
| Ginsburg et al., 2021 | Internal migration and health in South Africa: determinants of healthcare utilisation in a young adult cohort | Migration | Process-specific: Migration experience | Living outside of the study area at the time of interview | Momentary | Study area | Individual | Household census | None mentioned |
| Chawhanda et al., 2023 | Factors associated with access to condoms and HIV services among women in high migration communities in six Southern African countries | Migration | Process-specific: Migration experience | Migrant | Unknown | Unknown | Individual | Survey questionnaire | None mentioned |
| Bwambale et al., 2021 | Utilisation of sexual and reproductive health services among street children and young adults in Kampala, Uganda: does migration matter? | Migration | Process-specific: Migration experience | Migrants: moved from rural area to Kampala | Lifetime | Subnational region | Individual | Survey questionnaire | None mentioned |
| Koss et al., 2020 | Uptake, engagement, and adherence to pre-exposure prophylaxis offered after population HIV testing in rural Kenya and Uganda: 72-week interim analysis of observational data from the SEARCH study | Migration | Process-specific: Migration experience | Migrated outside of the community for at least 1 month or moved residence within the past 12 months | Past 12 months | Combination: Community if temporarily moved for a month or more, Usual residence if moved permanently | Individual | Survey questionnaire | None mentioned |
| Camlin et al., 2017 | High mobility and HIV prevalence among female market traders in East Africa in 2014 | Migration | Process-specific: Migration experience | Migration | Ever | Usual residence | Individual | Survey questionnaire | None mentioned |
| Kadede et al., 2016 | Increasing adolescent HIV testing with a hybrid mobile strategy in uganda and Kenya | Travel | Minimum period of time away | Number (1-6) months spent away from community in the past year | Past 12 months | Community | Individual | Survey questionnaire | None mentioned |
| Thorp et al., 2022 | Mobility and ART retention among men in Malawi: a mixed-methods study | Travel | Minimum period of time away | Mobile: spent at least 14 total nights away from home in the past 12 months With a long trip: at least 14 nights long (consecutive nights) Without a long trip | Past 12 months | Usual residence | Individual | Survey questionnaire | None mentioned |
| Reidy et al., 2018 | Engagement in care and infant HIV testing among lost to follow-up option B+ patients | Migration | Process-specific: Migration experience | Mother moved out of Eswatini | Since 6 months postpartum | Country | Individual, mother | EMR/routine health record/program data, patient tracing in the community | None mentioned |
| Onoya et al., 2021 | Understanding the Reasons for Deferring ART Among Patients Diagnosed Under the Same‐Day‐ART Policy in Johannesburg, South Africa | Migration | Process-specific: Migration experience | Moved from house | Past 6 months | Usual residence | Individual | Survey questionnaire | None mentioned |
| Olawore et al., 2018 | Migration and risk of HIV acquisition in Rakai, Uganda: a population-based cohort study | Migration | Process-specific: Migration experience | Moved into an RCCS community | Since last survey round | Study area | Individual | Household census | None mentioned |
| Brophy et al., 2021 | Prevalence of Untreated HIV and Associated Risk Behaviors Among the Sexual Partners of Recent Migrants and Long-term Residents in Rakai, Uganda | Migration | Process-specific: Migration experience | Moved into an RCCS community in the past 2 years | Past 24 months | Study area | Individual, Sexual network | Household census, Survey questionnaire including sexual partner block | None mentioned |
| Floyd et al., 2020 | HIV testing and treatment coverage achieved after 4 years across 14 urban and peri-urban communities in Zambia and South Africa: An analysis of findings from the HPTN 071 (PopART) trial | Migration | Process-specific: Migration experience | Moved into Community HIV Care Provider (CHiP) zone | Since Round 1 or 2 of the intervention | Community | Individual | Residency information recorded during follow up visits in the trial | None mentioned |
| Larmarange et al., 2018 | The impact of population dynamics on the population HIV care cascade: results from the ANRS 12249 Treatment as Prevention trial in rural KwaZulu-Natal (South Africa) | Migration | Process-specific: Migration experience | Moved into or out of the study area | Since last survey round | Study area | Individual | Household census | None mentioned |
| Kim et al., 2020 | Migration and first-year maternal mortality among HIV-positive postpartum women: A population-based longitudinal study in rural South Africa | Migration | Process-specific: Migration experience | Moved into or out of the study area during the pregnancy and first-year postpartum period | During pregnancy and first year postpartum | Study area | Individual | Household census | None mentioned |
| Bernardo et al., 2021 | Patterns of mobility and its impact on retention in care among people living with HIV in the Manhica District, Mozambique | Migration | Process-specific: Migration experience | Moved outside of Manhica District in the past 12 months | Past 12 months | Subnational region | Individual | Survey questionnaire | None mentioned |
| Etoori et al., 2020 | Outcomes After Being Lost to Follow-up Differ for Pregnant and Postpartum Women When Compared With the General HIV Treatment Population in Rural South Africa | Migration | Process-specific: Migration experience | Moved outside the study area since last clinic visit, excluding those who transferred clinics | Since last clinic visit | Study area | Individual | Household census | None mentioned |
| Dzomba et al., 2022 | Predictors of migration in an HIV hyper-endemic rural South African community: evidence from a population-based cohort (2005-2017) | Migration | Process-specific: Migration experience | Moved outside the surveillance area at least once | Since last survey round | Study area | Individual | Household census | None mentioned |
| Dzomba et al., 2019 | Effect of ART scale-up and female migration intensity on risk of HIV acquisition: results from a population-based cohort in KwaZulu-Natal, South Africa | Migration | Process-specific: Migration experience | Moved outside the surveillance area for at least 4 months at least once | Past 12 months | Study area | Individual | Household census | None mentioned |
| Slabbert et al., 2017 | Sexual and reproductive health outcomes among female sex workers in Johannesburg and Pretoria, South Africa: Recommendations for public health programmes | Migration | Process-specific: Migration experience | Moved residence at least once in the past year | Past 12 months | Usual residence | Individual | Survey questionnaire | None mentioned |
| Grabowski et al., 2020 | Migration, hotspots, and dispersal of HIV infection in Rakai, Uganda | Migration | Process-specific: Migration experience | Moved to a household in a new community within the study area, or moved to another community outside the study area | Since last survey round | Community | Individual, community, subnational region | Household census | None mentioned |
| Baisley et al., 2018 | High HIV incidence and low uptake of HIV prevention services: The context of risk for young male adults prior to DREAMS in rural KwaZulu-Natal, South Africa | Migration | Process-specific: Migration experience | Moving away from the surveillance area and subsequently returning to the surveillance area | Past 12 months | Study area | Individual | Household census | None mentioned |
| Chimbindi et al., 2018 | Persistently high incidence of HIV and poor service uptake in adolescent girls and young women in rural KwaZulu-Natal, South Africa prior to DREAMS | Migration | Process-specific: Migration experience | Moving away from the surveillance area and subsequently returning to the surveillance area | Past 12 months | Study area | Individual | Household census | None mentioned |
| Shearer et al., 2017 | Citizenship status and engagement in HIV care: An observational cohort study to assess the association between reporting a national ID number and retention in public-sector HIV care in Johannesburg, South Africa | Migration | Person-specific | No South African citizenship status:  1) Foreign national (citizenship elsewhere) 2) South African with unconfirmed citizenship status | Momentary | Country | Individual | EMR/routine health record/program data | For participants self-reporting South African nationality but lacking citizenship information in the routine health record were classified as "unconfirmed South African citizens" instead of non-citizens. |
| Fennell et al., 2023 | The impact of free antiretroviral therapy for pregnant non-citizens and their infants in Botswana | Migration | Person-specific | Non-citizen: country of origin not Botswana in the surveillance system | Lifetime | Country | Individual, mother | EMR/routine health record/program data | None mentioned |
| Chamie et al., 2016 | A hybrid mobile approach for population-wide HIV testing in rural east Africa: An observational study | Travel | Minimum period of time away | Spent 1-6 months away from community in the past 12 months | Past 12 months | Community | Individual | Survey questionnaire | None mentioned |
| Rosenberg et al., 2018 | Individual, Partner, and Couple Predictors of HIV Infection among Pregnant Women in Malawi: A Case-Control Study | Migration | Process-specific: Length of residence | Number of years living in current residence | Momentary | Usual residence | Individual, Sexual partner | Survey questionnaire | None mentioned |
| Agadjanian et al., 2021 | Men's migration and women's mortality in rural Mozambique | Migration | Process-specific: Migration experience | Out-migration | Since 2006 | Unknown | Marital partner | Survey questionnaire | None mentioned |
| Murnane et al., 2022 | Distinct forms of migration and mobility are differentially associated with HIV treatment adherence | Travel | Minimum period of time away | Overnight travel | Past 6 months | Usual residence | Individual | Survey questionnaire | None mentioned |
| Odimegwu et al., 2022 | A multilevel mixed effect analysis of neighbourhood and individual level determinants of risky sexual behaviour among young people in South Africa. | Migration | Process-specific: Migration experience | Percentage of residents in a community who have moved from current place of residence in the past five years ("residential instability") | Past 5 years | Usual residence | Community | Survey questionnaire | None mentioned |
| Camlin et al., 2019 | Gendered dimensions of population mobility associated with HIV across three epidemics in rural Eastern Africa | Migration | Process-specific: Migration experience | Permanent change of primary residence over national or subnational boundaries | Ever | Subnational region | Individual | Survey questionnaire with lifeline-method migration histories | None mentioned |
| Ambia et al., 2019 | Outcomes of patients lost to follow-up after antiretroviral therapy initiation in rural north-eastern South Africa | Migration | Process-specific: Migration experience | Permanent out-migration from the study area | Since last survey round | Study area | Individual | Household census | None mentioned |
| Coetzee et al., 2017 | Cross-sectional study of female sex workers in Soweto, South Africa: Factors associated with HIV infection | Migration | Person-specific | Place of birth not Gauteng province | Lifetime | Subnational region | Individual | Survey questionnaire | None mentioned |
| Marukutira et al., 2019-B | Comparison of knowledge of HIV status and treatment coverage between non-citizens and citizens: Botswana Combination Prevention Project (BCPP) | Migration | Person-specific | Self-reported no Botswana citizenship ("non-citizen") | Momentary | Country | Individual | Survey questionnaire | None mentioned |
| Low et al., 2019-B | Correlates of HIV infection in adolescent girls and young women in Lesotho: results from a population-based survey | Travel | Minimum period of time away | Away from the country for a month or more | Lifetime | Country | Individual | Survey questionnaire | None mentioned |
| Bulstra et al., 2020 | Mapping and characterising areas with high levels of HIV transmission in sub-Saharan Africa: A geospatial analysis of national survey data | Travel | Minimum period of time away | Slept in the house the night before (de facto resident) but not a usual resident (de jure) | Past night | Usual residence | Individual | Household census | None mentioned |
| Psaki et al., 2022 | What are we learning about HIV testing in informal settlements in KwaZulu-Natal, South Africa? Results from a randomized controlled trial | Travel | Minimum period of time away | Slept away from home for over 30 days in the past 6 months | Past 6 months | Usual residence | Individual | Survey questionnaire | None mentioned |
| Tomita et al., 2019 | Sociobehavioral and community predictors of unsuppressed HIV viral load: Multilevel results from a hyperendemic rural South African population | Migration | Process-specific: Migration experience | Spent 50% or more time outside the surveillance area prior to HIV acquisition | Lifetime period up until HIV acquisition | Study area | Individual | Household census | None mentioned |
| Vandormael et al., 2020 | HIV incidence declines in a rural South African population: a G-imputation approach for inference | Migration | Process-specific: Migration experience | Spent at least 5% of cumulative time residing outside the study area | Unknown | Study area | Individual | Household census | None mentioned |
| Camlin et al., 2017 | High mobility and HIV prevalence among female market traders in East Africa in 2014 | Travel | Minimum period of time away | Spent at least one night away from usual residence | Past 3 months | Usual residence | Individual | Survey questionnaire | None mentioned |
| Camlin et al., 2019 | Gendered dimensions of population mobility associated with HIV across three epidemics in rural Eastern Africa | Travel | Minimum period of time away | Spent at least one night away from usual residence | Past 3 months | Usual residence | Individual | Survey questionnaire with lifeline-method migration histories | None mentioned |
| Petersen et al., 2021 | Geographic mobility and time to seeking care among people with TB in Limpopo, South Africa | Travel | Minimum period of time away | Spent more than 30 nights at a residence other than the primary residence in the past 12 months, or were over 250 km away from primary residence at time of interview | Past 12 months | Usual residence | Individual | Survey questionnaire | None mentioned |
| Gorin et al., 2023 | Mobility and HIV vulnerabilities among female sex workers in Guinea-Bissau: findings from an integrated bio-behavioral survey | Travel | Minimum period of time away | Spent at least one night away from usual residence | Past 6 months | Usual residence | Individual | Survey questionnaire | None mentioned |
| Tomita et al., 2017 | Social Disequilibrium and the Risk of HIV Acquisition: A Multilevel Study in Rural KwaZulu-Natal Province, South Africa | Migration | Process-specific: Migration experience | Spent time residing the study area | Unknown | Study area | Individual, neighborhood | Household census | None mentioned |
| Edwards et al., 2019 | The HIV care continuum among resident and non-resident populations found in venues in East Africa cross-border areas | Travel | Minimum period of time away | Time spent away from primary residence in the past year two weeks or less 2-4 weeks 1-3 months 3+ months | Past 12 months | Usual residence | Individual | Survey questionnaire | None mentioned |
| Nakiire et al., 2020 | Factors Associated with Utilisation of Couple HIV Counselling and Testing Among HIV-Positive Adults in Kyoga Fishing Community Uganda, May 2017: Cross Sectional Study | Travel | Minimum period of time away | Traveled away and did not sleep at landing site in past 12 months | Past 12 months | Workplace | Individual | Survey questionnaire | None mentioned |
| Wahome et al., 2022 | Stopping and restarting PrEP and loss to follow-up among PrEP-taking men who have sex with men and transgender women at risk of HIV-1 participating in a prospective cohort study in Kenya | Travel | Movement between geographies | Traveled in the past 3 months | Past 3 months | Unknown | Individual | Survey questionnaire | None mentioned |
| Davey et al., 2019 | Mobility and sex work: why, where, when? A typology of female-sex-worker mobility in Zimbabwe | Travel | Movement between geographies | Worked or stayed elsewhere in the past 12 months | Past 12 months | Study area | Individual, Study area | Survey questionnaire including travel histories | None mentioned |
| Dememew et al., 2020 | The yield of community-based tuberculosis and HIV among key populations in hotspot settings of Ethiopia: A cross-sectional implementation study | Migration | Process-specific: Migration experience | Working in road construction or mining and identified as a migrant ("internal migratory worker") | Momentary | Unknown | Individual | Registration of internal migratory workers at workplaces | None mentioned |

## Table S4. Study-specific mobility metrics by dimension

| **Reference** | **Title** | **Spatial boundaries** | **Spatial distance** | **Features of the origin-destination locations** | **Frequency/ intensity** | **Duration** | **Social** | **Other** |
| --- | --- | --- | --- | --- | --- | --- | --- | --- |
| Nhampossa et al., 2021 | The impact of the caregiver mobility on child HIV care in the Manhica District, Southern Mozambique: A clinical based study | Internal, international |  | Destination location | Number of trips | At least 3 mobility events at least 4 nights long; Amount of time spent at destination | Work/business/looking for opportunity, visiting/supporting relatives, following the partner, religious ceremonies, other; Moved with child; Destination is a family house, own house, rented house, or other | Traveled with a passport |
| Low et al., 2021 | Migration in Namibia and its association with HIV acquisition and treatment outcomes | Inter-community, international |  |  |  | At least one mobility event over one month long; Recency of travel/migration |  |  |
| Bahemuka et al., 2023 | Factors Associated with Short and Long Term Mobility and HIV Risk of Women Living in Fishing Communities Around Lake Victoria in Kenya, Tanzania, and Uganda: A Cross Sectional Survey |  |  | Type of destination location (fishing community, village, town) | Number of trips | Amount of time spent away on most recent trip | Trading, visiting, going home, seeking healthcare, more than one reason, other |  |
| Goodman et al., 2016 | Child-street migration among HIV-affected families in Kenya: a mediation analysis from cross-sectional data. |  |  |  |  |  |  |  |
| Odayar et al., 2023 | Mobility during the post-partum period and viraemia in women living with HIV in South Africa |  |  | Destination location | Number of trips | At least one night spent away; Total time spent away |  | Attended a health facility at destination |
| Nyabuti et al., 2021 | Characteristics of HIV seroconverters in the setting of universal test and treat: Results from the SEARCH trial in rural Uganda and Kenya |  |  |  |  |  |  |  |
| Shaw et al., 2023 | Geographical Associations of HIV Prevalence in Female Sex Workers From Nairobi, Kenya (2014-2017) |  |  |  |  |  |  |  |
| Marukutira et al., 2019-A | Clinical outcomes of a cohort of migrants and citizens living with human immunodeficiency virus in Botswana: Implications for Joint United Nation Program on HIV and AIDS 90-90-90 targets |  |  |  |  |  |  |  |
| Murnane et al., 2022 | Distinct forms of migration and mobility are differentially associated with HIV treatment adherence | Intra-region, inter-region, international |  |  |  | Recency of travel/migration | For opportunity (job, education, improved housing), after loss (divorce, death of a spouse, fleeing violence, loss of employment), for family (marriage, accompanying family member, to care or be care for by family member) | Age at the time of migration |
| Dobra et al., 2017 | Space-time migration patterns and risk of HIV acquisition in rural South Africa | Inter-community, intra-community | Sum of the distances between consecutive residences per year | In vs. out migration | Number of migration events | Percentage of time spent living outside current location |  |  |
| Dobra et al., 2019 | A method for statistical analysis of repeated residential movements to link human mobility and HIV acquisition | Intra-community, inter-community; Within vs. outside study area |  | Destination location | Number of migration events |  |  |  |
| Kiyingi et al., 2023 | Predictors of mobility among women engaged in commercial sex work in Uganda using generalized estimating equations model |  |  |  |  | Recency of travel/migration |  |  |
| Dorward et al., 2017 | Factors associated with poor linkage to HIV care in South Africa: Secondary analysis of data from the Thol'impilo trial |  |  |  |  |  |  |  |
| Beres et al., 2021 | Patterns and Predictors of Incident Return to HIV Care Among Traced, Disengaged Patients in Zambia: Analysis of a Prospective Cohort |  |  |  |  | At least one mobility event over one month long |  |  |
| Camlin et al., 2017 | High mobility and HIV prevalence among female market traders in East Africa in 2014 |  |  |  |  | Number of years living in current location |  |  |
| Davey et al., 2020 | Exploring the Association Between Mobility and Access to HIV Services Among Female Sex Workers in Zimbabwe | Internal, international |  | Origin/destination linguistic area; Availability of the program clinic at the destination |  | At least one day spent away | For sex work | Whether healthcare was used at the destination; Whether worked in sex work at destination |
| Lee et al., 2023 | Condom, modern contraceptive, and dual method use are associated with HIV status and relationship concurrency in a context of high mobility: A cross-sectional study of women of reproductive age in rural Kenya and Uganda, 2016 |  |  |  |  |  |  |  |
| Mthiyane et al., 2022 | The association of exposure to DREAMS on sexually acquiring or transmitting HIV amongst adolescent girls and young women living in rural South Africa |  |  |  |  |  |  |  |
| Senteza et al., 2023 | Virological non-suppression among adult males attending HIV care services in the fishing communities in Bulisa district, Uganda |  |  |  | Number of trips |  |  |  |
| Janse Van Rensburg et al., 2021 | Healthcare without borders: A cross-sectional study of immigrant and nonimmigrant children admitted to a large public sector hospital in the Gauteng Province of South Africa |  |  |  |  |  |  |  |
| Grabowski et al., 2021 | Prevalence and Predictors of Persistent Human Immunodeficiency Virus Viremia and Viral Rebound after Universal Test and Treat: A Population-Based Study |  |  |  |  |  |  |  |
| Billioux et al., 2017 | Human immunodeficiency virus care cascade among sub-populations in Rakai, Uganda: an observational study. |  |  |  |  |  |  |  |
| Plymoth et al., 2020 | Socio-economic condition and lack of virological suppression among adults and adolescents receiving antiretroviral therapy in Ethiopia |  |  | Number of unique destination locations; Job location at destination |  |  |  |  |
| Correa-Agudelo et al., 2021 | Associated health and social determinants of mobile populations across HIV epidemic gradients in Southern Africa. |  |  |  |  |  |  |  |
| Psaki et al., 2022 | What are we learning about HIV testing in informal settlements in KwaZulu-Natal, South Africa? Results from a randomized controlled trial |  |  |  |  | Number of years living in current location |  |  |
| Low et al., 2019-A | Association between severe drought and HIV prevention and care behaviors in Lesotho: A population-based survey 2016-2017 |  |  |  |  |  |  |  |
| Ginsburg et al., 2021 | Internal migration and health in South Africa: determinants of healthcare utilisation in a young adult cohort |  |  |  |  |  |  |  |
| Chawhanda et al., 2023 | Factors associated with access to condoms and HIV services among women in high migration communities in six Southern African countries | Internal, international |  |  |  |  |  |  |
| Bwambale et al., 2021 | Utilisation of sexual and reproductive health services among street children and young adults in Kampala, Uganda: does migration matter? |  |  | Rural-to-urban migration |  | Recency of travel/migration |  |  |
| Koss et al., 2020 | Uptake, engagement, and adherence to pre-exposure prophylaxis offered after population HIV testing in rural Kenya and Uganda: 72-week interim analysis of observational data from the SEARCH study |  |  |  |  | At least one mobility event over one month long |  |  |
| Camlin et al., 2017 | High mobility and HIV prevalence among female market traders in East Africa in 2014 |  |  |  |  | Recency of travel/migration | Business/earning income, marriage, to accompany husband or other family member, sent by family, to care or be cared for by family member, to flee from violence, education, other |  |
| Kadede et al., 2016 | Increasing adolescent HIV testing with a hybrid mobile strategy in uganda and Kenya |  |  |  |  | Spent at least one month away; Cumulative time spent away |  |  |
| Thorp et al., 2022 | Mobility and ART retention among men in Malawi: a mixed-methods study | Intra-region, inter-region, international |  |  |  | At least one trip over 14 nights long; Spent at least 14 nights cumulatively away |  |  |
| Reidy et al., 2018 | Engagement in care and infant HIV testing among lost to follow-up option B+ patients |  |  |  |  |  |  |  |
| Onoya et al., 2021 | Understanding the Reasons for Deferring ART Among Patients Diagnosed Under the Same‐Day‐ART Policy in Johannesburg, South Africa | Intra-regional, inter-regional, international |  |  |  |  |  |  |
| Olawore et al., 2018 | Migration and risk of HIV acquisition in Rakai, Uganda: a population-based cohort study |  |  |  |  | Number of years living in current location |  |  |
| Brophy et al., 2021 | Prevalence of Untreated HIV and Associated Risk Behaviors Among the Sexual Partners of Recent Migrants and Long-term Residents in Rakai, Uganda |  |  |  |  |  |  |  |
| Floyd et al., 2020 | HIV testing and treatment coverage achieved after 4 years across 14 urban and peri-urban communities in Zambia and South Africa: An analysis of findings from the HPTN 071 (PopART) trial |  |  |  |  |  |  |  |
| Larmarange et al., 2018 | The impact of population dynamics on the population HIV care cascade: results from the ANRS 12249 Treatment as Prevention trial in rural KwaZulu-Natal (South Africa) |  |  |  |  |  |  |  |
| Kim et al., 2020 | Migration and first-year maternal mortality among HIV-positive postpartum women: A population-based longitudinal study in rural South Africa | Within vs. outside study area |  |  |  |  |  | Delivered in origin/destination location |
| Bernardo et al., 2021 | Patterns of mobility and its impact on retention in care among people living with HIV in the Manhica District, Mozambique | Internal, international |  |  |  |  |  |  |
| Etoori et al., 2020 | Outcomes After Being Lost to Follow-up Differ for Pregnant and Postpartum Women When Compared With the General HIV Treatment Population in Rural South Africa |  |  |  |  |  |  |  |
| Dzomba et al., 2022 | Predictors of migration in an HIV hyper-endemic rural South African community: evidence from a population-based cohort (2005-2017) |  |  |  | Number of migration events; Incidence of migration events |  |  |  |
| Dzomba et al., 2019 | Effect of ART scale-up and female migration intensity on risk of HIV acquisition: results from a population-based cohort in KwaZulu-Natal, South Africa |  |  |  | Number of migrations events | At least one mobility event over 4 months long |  |  |
| Slabbert et al., 2017 | Sexual and reproductive health outcomes among female sex workers in Johannesburg and Pretoria, South Africa: Recommendations for public health programmes |  |  |  |  |  |  |  |
| Grabowski et al., 2020 | Migration, hotspots, and dispersal of HIV infection in Rakai, Uganda |  | Distance between origin and destination | Origin location; Origin/destination location is a region with high in-migration rate (inland sub-district, Tanzania, Kampala, or Masaka district); In vs. out-migration | Number of in-migrants per year; In-migration rate per 100 person-years; Out-migration rate per 100 person-years | Amount of time since arrival | Newly married, divorced/separated, work, starting a new household, living with friends/relatives |  |
| Baisley et al., 2018 | High HIV incidence and low uptake of HIV prevention services: The context of risk for young male adults prior to DREAMS in rural KwaZulu-Natal, South Africa |  |  |  |  |  |  |  |
| Chimbindi et al., 2018 | Persistently high incidence of HIV and poor service uptake in adolescent girls and young women in rural KwaZulu-Natal, South Africa prior to DREAMS |  |  |  |  |  |  |  |
| Shearer et al., 2017 | Citizenship status and engagement in HIV care: An observational cohort study to assess the association between reporting a national ID number and retention in public-sector HIV care in Johannesburg, South Africa |  |  |  |  |  |  |  |
| Fennell et al., 2023 | The impact of free antiretroviral therapy for pregnant non-citizens and their infants in Botswana |  |  |  |  |  |  |  |
| Chamie et al., 2016 | A hybrid mobile approach for population-wide HIV testing in rural east Africa: An observational study |  |  |  |  | Spent at least one month away; Cumulative time spent away |  |  |
| Rosenberg et al., 2018 | Individual, Partner, and Couple Predictors of HIV Infection among Pregnant Women in Malawi: A Case-Control Study |  |  |  |  | Number of years living in current location |  |  |
| Agadjanian et al., 2021 | Men's migration and women's mortality in rural Mozambique |  |  |  |  |  |  | Frequency of remittances; Perceived migration success (improved household conditions) |
| Murnane et al., 2022 | Distinct forms of migration and mobility are differentially associated with HIV treatment adherence |  |  |  | Number of trips | Average time spent away | Work, Non-work |  |
| Odimegwu et al., 2022 | A multilevel mixed effect analysis of neighbourhood and individual level determinants of risky sexual behaviour among young people in South Africa. |  |  |  |  |  |  |  |
| Camlin et al., 2019 | Gendered dimensions of population mobility associated with HIV across three epidemics in rural Eastern Africa | intra-regional, inter-regional, international |  |  |  | Recency of travel/migration |  |  |
| Ambia et al., 2019 | Outcomes of patients lost to follow-up after antiretroviral therapy initiation in rural north-eastern South Africa |  |  |  |  |  |  |  |
| Coetzee et al., 2017 | Cross-sectional study of female sex workers in Soweto, South Africa: Factors associated with HIV infection | Internal, international |  |  |  |  |  |  |
| Marukutira et al., 2019-B | Comparison of knowledge of HIV status and treatment coverage between non-citizens and citizens: Botswana Combination Prevention Project (BCPP) |  |  |  |  |  |  |  |
| Low et al., 2019-B | Correlates of HIV infection in adolescent girls and young women in Lesotho: results from a population-based survey |  |  |  |  | Recency of travel/migration |  |  |
| Bulstra et al., 2020 | Mapping and characterising areas with high levels of HIV transmission in sub-Saharan Africa: A geospatial analysis of national survey data |  |  |  |  |  |  |  |
| Psaki et al., 2022 | What are we learning about HIV testing in informal settlements in KwaZulu-Natal, South Africa? Results from a randomized controlled trial |  |  |  |  | Spent at least 30 days away |  |  |
| Tomita et al., 2019 | Sociobehavioral and community predictors of unsuppressed HIV viral load: Multilevel results from a hyperendemic rural South African population |  |  |  |  | Spent at least 50% of time living outside current location |  |  |
| Vandormael et al., 2020 | HIV incidence declines in a rural South African population: a G-imputation approach for inference |  |  |  |  | Spent at least 5% of time living outside current location |  |  |
| Camlin et al., 2017 | High mobility and HIV prevalence among female market traders in East Africa in 2014 |  |  |  |  | At least one night spent away; Recency of travel/migration |  |  |
| Camlin et al., 2019 | Gendered dimensions of population mobility associated with HIV across three epidemics in rural Eastern Africa |  |  |  | Number of trips | Spent at least one night away; Average time spent away; Recency of travel/migration | Labor-related (artisanal, farming, fishing, looking for work, market trading), Non-work travel (caregiving/careseeking, funeral, holiday/visiting family, other, schooling) |  |
| Petersen et al., 2021 | Geographic mobility and time to seeking care among people with TB in Limpopo, South Africa |  | Distance away from usual residence at the time of interview; Maximum distance traveled |  |  | At least one mobility event over one month long; Total time spent away; Number of years living in current location |  | If became sick away from home, would return home/seek care while away/not seek care |
| Gorin et al., 2023 | Mobility and HIV vulnerabilities among female sex workers in Guinea-Bissau: findings from an integrated bio-behavioral survey | Internal, international |  | Number of unique destination locations; Destination location; Residence at destination |  | At least one night spent away |  |  |
| Tomita et al., 2017 | Social Disequilibrium and the Risk of HIV Acquisition: A Multilevel Study in Rural KwaZulu-Natal Province, South Africa |  |  |  |  | Percentage of time spent living outside current location |  |  |
| Edwards et al., 2019 | The HIV care continuum among resident and non-resident populations found in venues in East Africa cross-border areas |  |  |  |  | Total time spent away |  |  |
| Nakiire et al., 2020 | Factors Associated with Utilisation of Couple HIV Counselling and Testing Among HIV-Positive Adults in Kyoga Fishing Community Uganda, May 2017: Cross Sectional Study |  |  |  |  |  |  |  |
| Wahome et al., 2022 | Stopping and restarting PrEP and loss to follow-up among PrEP-taking men who have sex with men and transgender women at risk of HIV-1 participating in a prospective cohort study in Kenya |  |  |  |  |  |  |  |
| Davey et al., 2019 | Mobility and sex work: why, where, when? A typology of female-sex-worker mobility in Zimbabwe |  | Median distance between origin and destination; Median circuit distance | Number of destination locations; median number of destination locations; type of destination location (town/city, growth point, or mine/farm) | Number of trips | Total time spent away | Work, non-work |  |
| Dememew et al., 2020 | The yield of community-based tuberculosis and HIV among key populations in hotspot settings of Ethiopia: A cross-sectional implementation study |  |  |  |  |  |  |  |

## Table S5. Study-specific research contexts

| **Reference** | **Title** | **Data source** | **Dates of data collection** | **Study design** | **Study population** | **Country/ Countries** | **Geographic context** | **HIV Domain** | **Relevant GAM indicator(s)** | **HIV Indicator(s)** |
| --- | --- | --- | --- | --- | --- | --- | --- | --- | --- | --- |
| Agadjanian et al., 2021 | Men's migration and women's mortality in rural Mozambique | Men's Migrations and Women's Lives (MMWL) | 2006 to 2018 | Cohort | Women | Mozambique | Rural/agricultural | Epidemiology | 2.7 AIDS mortality | AIDS-related death |
| Murnane et al., 2022 | Distinct forms of migration and mobility are differentially associated with HIV treatment adherence | SEARCH | February to November 2016 | Cross-sectional | Adults | Kenya, Uganda | Rural/agricultural, Fishing villages, Border areas | Care and treatment | 2.2 People living with HIV on antiretroviral therapy | ART adherence |
| Murnane et al., 2022 | Distinct forms of migration and mobility are differentially associated with HIV treatment adherence | SEARCH | February to November 2016 | Cross-sectional | Adults | Kenya, Uganda | Rural/agricultural, Fishing villages, Border areas | Care and treatment | 2.2 People living with HIV on antiretroviral therapy | ART adherence |
| Nhampossa et al., 2021 | The impact of the caregiver mobility on child HIV care in the Manhica District, Southern Mozambique: A clinical based study | Primary | December 2017 to February 2018 | Cross-sectional | Children | Mozambique | Rural/agricultural | Care and treatment | 2.2 People living with HIV on antiretroviral therapy | ART adherence, Retention in care |
| Odayar et al., 2023 | Mobility during the post-partum period and viraemia in women living with HIV in South Africa | Primary | January 2016 to December 2019 | Cohort | Post-partum women | South Africa | Urban/trading center | Care and treatment | 2.2 People living with HIV on antiretroviral therapy; 2.3 People living with HIV who have suppressed viral loads | ART adherence, Viral suppression |
| Etoori et al., 2020 | Outcomes After Being Lost to Follow-up Differ for Pregnant and Postpartum Women When Compared With the General HIV Treatment Population in Rural South Africa | Agincourt HDSS | 2014 to 2017 | Cohort | Adults | South Africa | Rural/agricultural, Border area | Care and treatment, PMTCT | 2.2 People living with HIV on antiretroviral therapy; 3.1 Preventing vertical transmission of HIV | ART coverage, PMTCT coverage, Retention in care |
| Marukutira et al., 2019-A | Clinical outcomes of a cohort of migrants and citizens living with human immunodeficiency virus in Botswana: Implications for Joint United Nation Program on HIV and AIDS 90-90-90 targets | EMR/Program data | January 2002 to December 2016 | Retrospective Cohort | Adults and children | Botswana | Urban/trading center | Epidemiology, Care and treatment | 2.2 People living with HIV on antiretroviral therapy; 2.3 People living with HIV who have suppressed viral loads; 2.7 AIDS mortality | ART coverage, Viral suppression, Retention in care, AIDS-related deaths |
| Ambia et al., 2019 | Outcomes of patients lost to follow-up after antiretroviral therapy initiation in rural north-eastern South Africa | ACDIS Cohort | April 2014 to July 2017 | Cohort | Adults | South Africa | Rural/agricultural, Border area | Care and treatment | 2.2 People living with HIV on antiretroviral therapy | ART initiation |
| Onoya et al., 2021 | Understanding the Reasons for Deferring ART Among Patients Diagnosed Under the Same‐Day‐ART Policy in Johannesburg, South Africa | Primary | October 2017 to August 2018 | Cohort | Adults | South Africa | Urban/trading center | Care and treatment | 2.2 People living with HIV on antiretroviral therapy | ART initiation, ART adherence |
| Odimegwu et al., 2022 | A multilevel mixed effect analysis of neighbourhood and individual level determinants of risky sexual behaviour among young people in South Africa. | DHS | 2016 | Cross-sectional | Adolescents and young people | South Africa | National | Prevention | 1.14 Condom use at last high-risk sex | Condom use |
| Bahemuka et al., 2023 | Factors Associated with Short and Long Term Mobility and HIV Risk of Women Living in Fishing Communities Around Lake Victoria in Kenya, Tanzania, and Uganda: A Cross Sectional Survey | Primary | 2018 to 2019 | Cross-sectional | Women | Kenya, Tanzania, Uganda | Fishing villages | Prevention | 1.14 Condom use at last high-risk sex | Condom use |
| Lee et al., 2023 | Condom, modern contraceptive, and dual method use are associated with HIV status and relationship concurrency in a context of high mobility: A cross-sectional study of women of reproductive age in rural Kenya and Uganda, 2016 | SEARCH | February to November 2016 | Cross-sectional | Women | Kenya, Uganda | Rural/agricultural, Fishing villages, Border areas | Prevention | 1.14 Condom use at last high-risk sex | Condom use |
| Gorin et al., 2023 | Mobility and HIV vulnerabilities among female sex workers in Guinea-Bissau: findings from an integrated bio-behavioral survey | Primary | September 2017 to January 2018 | Cross-sectional | Female sex workers | Guinea-Bissau | Urban/trading center | Prevention | 1.14 Condom use at last high-risk sex | Condom use |
| Chawhanda et al., 2023 | Factors associated with access to condoms and HIV services among women in high migration communities in six Southern African countries | Sexual and Reproductive Health and Rights-HIV (SRHR-HIV) Knows no Borders Project | May to December 2018 | Cross-sectional | Women | Eswatini, Lesotho, Malawi, Mozambique, South Africa, Zambia | Transit corridor | Prevention | 1.14 Condom use at last high-risk sex; 2.5 HIV testing volume and positivity | Condom use, HIV testing |
| Dzomba et al., 2019 | Effect of ART scale-up and female migration intensity on risk of HIV acquisition: results from a population-based cohort in KwaZulu-Natal, South Africa | ACDIS Cohort | 2004 to 2015 | Cohort | Women | South Africa | Rural/agricultural, Border area | Epidemiology | 1.1 HIV incidence | HIV acquisition |
| Vandormael et al., 2020 | HIV incidence declines in a rural South African population: a G-imputation approach for inference | ACDIS Cohort | 2005 to 2018 | Cohort | Adults | South Africa | Rural/agricultural, Border area | Epidemiology | 1.1 HIV incidence | HIV acquisition |
| Tomita et al., 2017 | Social Disequilibrium and the Risk of HIV Acquisition: A Multilevel Study in Rural KwaZulu-Natal Province, South Africa | ACDIS Cohort | 2004 to 2015 | Cohort | Adults | South Africa | Rural/agricultural, Border area | Epidemiology | 1.1 HIV incidence | HIV acquisition |
| Nyabuti et al., 2021 | Characteristics of HIV seroconverters in the setting of universal test and treat: Results from the SEARCH trial in rural Uganda and Kenya | SEARCH | 2013 to 2017 | Cohort | Adults | Kenya, Uganda | Rural/agricultural, Fishing villages, Border areas | Epidemiology | 1.1 HIV incidence | HIV acquisition |
| Olawore et al., 2018 | Migration and risk of HIV acquisition in Rakai, Uganda: a population-based cohort study | RCCS | April 1999 to January 2015 | Cohort | Adults | Uganda | Rural/agricultural, Urban/trading center, Fishing villages, Border areas, Transit corridors | Epidemiology | 1.1 HIV incidence | HIV acquisition |
| Dobra et al., 2017 | Space-time migration patterns and risk of HIV acquisition in rural South Africa | ACDIS Cohort | January 2004 to December 2014 | Cohort | Adults | South Africa | Rural/agricultural, Border area | Epidemiology | 1.1 HIV incidence | HIV acquisition |
| Dobra et al., 2019 | A method for statistical analysis of repeated residential movements to link human mobility and HIV acquisition | ACDIS Cohort | January 2004 to December 2016 | Cohort | Adults | South Africa | Rural/agricultural, Border area | Epidemiology | 1.1 HIV incidence | HIV acquisition |
| Low et al., 2019-B | Correlates of HIV infection in adolescent girls and young women in Lesotho: results from a population-based survey | PHIA | November 2016 to May 2017 | Cross-sectional | Adolescent girls and young women | Lesotho | National | Epidemiology | 1.1 HIV incidence; HIV Prevalence | HIV acquisition, HIV serostatus |
| Low et al., 2021 | Migration in Namibia and its association with HIV acquisition and treatment outcomes | PHIA | June to December 2017 | Cross-sectional | Adults | Namibia | National | Epidemiology, Prevention, Care and treatment | 1.1 HIV incidence; HIV Prevalence; 2.1 People living with HIV who know their HIV status; 2.2 People living with HIV on antiretroviral therapy; 2.3 People living with HIV who have suppressed viral loads; 2.5 HIV testing volume and positivity | HIV acquisition, HIV serostatus, HIV testing, Known HIV positive status, ART coverage, ART adherence, Viral suppression |
| Chimbindi et al., 2018 | Persistently high incidence of HIV and poor service uptake in adolescent girls and young women in rural KwaZulu-Natal, South Africa prior to DREAMS | ACDIS Cohort | 2006 to 2015 | Cohort | Adolescent girls and young women | South Africa | Rural/agricultural, Border area | Epidemiology, Prevention | 1.1 HIV incidence; 2.5 HIV testing volume and positivity | HIV acquisition, HIV testing |
| Mthiyane et al., 2022 | The association of exposure to DREAMS on sexually acquiring or transmitting HIV amongst adolescent girls and young women living in rural South Africa | ACDIS Cohort | 2016 to 2018 | Cohort | Adolescent girls and young women | South Africa | Rural/agricultural, Border area | Epidemiology, Care and treatment | 1.1 HIV incidence; 2.3 People living with HIV who have suppressed viral loads | HIV acquisition, Viral suppression |
| Baisley et al., 2018 | High HIV incidence and low uptake of HIV prevention services: The context of risk for young male adults prior to DREAMS in rural KwaZulu-Natal, South Africa | ACDIS Cohort | 2006 to 2015 | Cohort | Young men | South Africa | Rural/agricultural, Border area | Epidemiology, Prevention | 1.1 HIV incidence; 1.12 Prevalence of male circumcision; 2.5 HIV testing volume and positivity | HIV acquisition, VMMC, HIV testing |
| Rosenberg et al., 2018 | Individual, Partner, and Couple Predictors of HIV Infection among Pregnant Women in Malawi: A Case-Control Study | Primary | December 2015 to December 2016 | Case-control | Pregnant women | Malawi | Urban/trading center | Epidemiology | HIV Prevalence | HIV serostatus |
| Slabbert et al., 2017 | Sexual and reproductive health outcomes among female sex workers in Johannesburg and Pretoria, South Africa: Recommendations for public health programmes | Primary | 2014 to 2015 | Cross-sectional | Female sex workers | South Africa | Urban/trading center | Epidemiology | HIV Prevalence | HIV serostatus |
| Petersen et al., 2021 | Geographic mobility and time to seeking care among people with TB in Limpopo, South Africa | Kharitode TB Study | July 2018 to January 2020 | Cross-sectional | Adults | South Africa | Rural/agricultural, Border area | Epidemiology | HIV Prevalence | HIV serostatus |
| Goodman et al., 2016 | Child-street migration among HIV-affected families in Kenya: a mediation analysis from cross-sectional data. | Primary | 2015 | Cross-sectional | Mothers or caregiving women and their children | Kenya | Rural/agricultural | Epidemiology | HIV Prevalence | HIV serostatus |
| Dememew et al., 2020 | The yield of community-based tuberculosis and HIV among key populations in hotspot settings of Ethiopia: A cross-sectional implementation study | Primary | August 2017 to January 2018 | Cross-sectional | Female sex workers, health care workers, prison inmates, homeless, internally displaced people, internal migratory workers and residents in missionary charities | Ethiopia | Rural/agricultural, Urban/trading center, mining town | Epidemiology | HIV Prevalence | HIV serostatus |
| Davey et al., 2019 | Mobility and sex work: why, where, when? A typology of female-sex-worker mobility in Zimbabwe | SAPPH-Ire trial | 2013 and 2016 | Repeated cross-sectional studies | Female sex workers | Zimbabwe | Urban/trading center, Rural/agricultural, Mining towns, Transit corridor | Epidemiology | HIV Prevalence | HIV serostatus |
| Kiyingi et al., 2023 | Predictors of mobility among women engaged in commercial sex work in Uganda using generalized estimating equations model | Primary | April 2019 to December 2022 | Longitudinal analysis within an RCT | Female sex workers | Uganda | Transit corridor | Epidemiology | HIV Prevalence | HIV serostatus |
| Coetzee et al., 2017 | Cross-sectional study of female sex workers in Soweto, South Africa: Factors associated with HIV infection | Primary | February to September 2016 | Cross-sectional | Female sex workers | South Africa | Urban/trading center | Epidemiology | HIV Prevalence | HIV serostatus |
| Camlin et al., 2017 | High mobility and HIV prevalence among female market traders in East Africa in 2014 | Primary | January to June 2014 | Cross-sectional | Female market traders | Kenya | Urban/trading center | Epidemiology | HIV Prevalence | HIV serostatus |
| Camlin et al., 2017 | High mobility and HIV prevalence among female market traders in East Africa in 2014 | Primary | January to June 2014 | Cross-sectional | Female market traders | Kenya | Urban/trading center | Epidemiology | HIV Prevalence | HIV serostatus |
| Camlin et al., 2017 | High mobility and HIV prevalence among female market traders in East Africa in 2014 | Primary | January to June 2014 | Cross-sectional | Female market traders | Kenya | Urban/trading center | Epidemiology | HIV Prevalence | HIV serostatus |
| Camlin et al., 2019 | Gendered dimensions of population mobility associated with HIV across three epidemics in rural Eastern Africa | SEARCH | February to November 2016 | Cohort | Adults | Kenya, Uganda | Rural/agricultural, Fishing villages, Border areas | Epidemiology | HIV Prevalence | HIV serostatus |
| Camlin et al., 2019 | Gendered dimensions of population mobility associated with HIV across three epidemics in rural Eastern Africa | SEARCH | February to November 2016 | Cohort | Adults | Kenya, Uganda | Rural/agricultural, Fishing villages, Border areas | Epidemiology | HIV Prevalence | HIV serostatus |
| Dzomba et al., 2022 | Predictors of migration in an HIV hyper-endemic rural South African community: evidence from a population-based cohort (2005-2017) | ACDIS Cohort | 2004 to 2017 | Cohort | Adults | South Africa | Rural/agricultural, Border area | Epidemiology | HIV Prevalence | HIV serostatus |
| Ginsburg et al., 2021 | Internal migration and health in South Africa: determinants of healthcare utilisation in a young adult cohort | The Migrant Health Follow-up Study (MHFUS) within the Agincourt HDSS | 2018 | Cross-sectional | Adults | South Africa | Rural/agricultural, Border area | Epidemiology | HIV Prevalence | HIV serostatus |
| Low et al., 2019-A | Association between severe drought and HIV prevention and care behaviors in Lesotho: A population-based survey 2016-2017 | PHIA | November 2016 to May 2017 | Cross-sectional | Adults | Lesotho | National | Epidemiology | HIV Prevalence | HIV serostatus |
| Bulstra et al., 2020 | Mapping and characterising areas with high levels of HIV transmission in sub-Saharan Africa: A geospatial analysis of national survey data | DHS | 2008 to 2016 | Cross-sectional | Adolescents and young people | Kenya, Malawi, Mozambique, Tanzania, Uganda, Zambia, Zimbabwe | National | Epidemiology | HIV Prevalence | HIV serostatus |
| Kim et al., 2020 | Migration and first-year maternal mortality among HIV-positive postpartum women: A population-based longitudinal study in rural South Africa | ACDIS Cohort | 2000 to 2016 | Cohort | Peri-to-postpartum women | South Africa | Rural/agricultural, Border area | Epidemiology | HIV Prevalence; 2.7 AIDS mortality | HIV serostatus, AIDS-related mortality |
| Grabowski et al., 2020 | Migration, hotspots, and dispersal of HIV infection in Rakai, Uganda | RCCS | August 2011 to January 2015 | Cohort | Adults | Uganda | Rural/agricultural, Urban/trading center, Fishing villages, Border areas, Transit corridors | Epidemiology, Care and treatment | HIV Prevalence; 2.2 People living with HIV on antiretroviral therapy | HIV serostatus, ART coverage |
| Marukutira et al., 2019-B | Comparison of knowledge of HIV status and treatment coverage between non-citizens and citizens: Botswana Combination Prevention Project (BCPP) | Botswana Combination Prevention Project (BCPP) | October 2013 to November 2015 | Cross-sectional | Adults | Botswana | Rural/agricultural, Urban/trading center, Border area | Epidemiology, Care and treatment | HIV Prevalence; 2.2 People living with HIV on antiretroviral therapy | HIV serostatus, ART coverage |
| Shaw et al., 2023 | Geographical Associations of HIV Prevalence in Female Sex Workers From Nairobi, Kenya (2014-2017) | EMR/Program data | 2014 to 2017 | Cross-sectional | Female sex workers | Kenya | Urban/trading center | Epidemiology, Prevention | HIV Prevalence; 1.14 Condom use at last high risk sex | HIV serostatus, Condom use |
| Correa-Agudelo et al., 2021 | Associated health and social determinants of mobile populations across HIV epidemic gradients in Southern Africa. | DHS | 2015 to 2018 | Cross-sectional | Adults | Angola, Malawi, South Africa, Zambia, Zimbabwe | National | Epidemiology, Prevention | HIV Prevalence; 1.14 Condom use at last high risk sex | HIV serostatus, Condom use |
| Fennell et al., 2023 | The impact of free antiretroviral therapy for pregnant non-citizens and their infants in Botswana | Tsepamo Surveillance Study | August 2014 to September 2021 | Pre-post | Pregnant to peri-partum women | Botswana | Rural/agricultural, Urban/trading center | Epidemiology | HIV Prevalence; 2.1 People living with HIV who know their HIV status; 2.7 AIDS mortality | HIV serostatus, Known HIV positive status, AIDS-related death |
| Floyd et al., 2020 | HIV testing and treatment coverage achieved after 4 years across 14 urban and peri-urban communities in Zambia and South Africa: An analysis of findings from the HPTN 071 (PopART) trial | HPTN 071 (PopART) trial | 2014 to 2017 | Longitudinal analysis within an RCT | Adults | Zambia | Urban/trading center, Border areas | Epidemiology, Care and treatment | HIV Prevalence; 2.1 People living with HIV who know their HIV status; 2.2 People living with HIV on antiretroviral therapy | HIV serostatus, Known HIV positive status, ART coverage |
| Janse Van Rensburg et al., 2021 | Healthcare without borders: A cross-sectional study of immigrant and nonimmigrant children admitted to a large public sector hospital in the Gauteng Province of South Africa | Primary | 2016 to 2017 | Cross-sectional | Children | South Africa | Urban/trading center | Epidemiology, PMTCT | HIV Prevalence; 3.1 Preventing vertical transmission of HIV | HIV serostatus, PMTCT coverage |
| Reidy et al., 2018 | Engagement in care and infant HIV testing among lost to follow-up option B+ patients | EMR/Program data | September 2013 to February 2015 | Longitudinal analysis of facility data | Mothers and their infants | Eswatini | Rural/agricultural, Urban/trading center | Epidemiology, PMTCT | HIV Prevalence; 3.1 Preventing vertical transmission of HIV | HIV serostatus, PMTCT coverage |
| Bwambale et al., 2021 | Utilisation of sexual and reproductive health services among street children and young adults in Kampala, Uganda: does migration matter? | Primary | May to July 2019 | Cross-sectional | Adolescents and young people | Uganda | Urban/trading center | Prevention | 2.5 HIV testing volume and positivity | HIV testing |
| Psaki et al., 2022 | What are we learning about HIV testing in informal settlements in KwaZulu-Natal, South Africa? Results from a randomized controlled trial | Primary | February 2017 to August 2019 | RCT | Adults | South Africa | Informal settlement | Prevention | 2.5 HIV testing volume and positivity | HIV testing |
| Psaki et al., 2022 | What are we learning about HIV testing in informal settlements in KwaZulu-Natal, South Africa? Results from a randomized controlled trial | Primary | February 2017 to August 2019 | RCT | Adults | South Africa | Informal settlement | Prevention | 2.5 HIV testing volume and positivity | HIV testing |
| Nakiire et al., 2020 | Factors Associated with Utilisation of Couple HIV Counselling and Testing Among HIV-Positive Adults in Kyoga Fishing Community Uganda, May 2017: Cross Sectional Study | HIV Behavioral survey | May 2017 | Cross-sectional | Adults | Uganda | Fishing villages | Prevention | 2.5 HIV testing volume and positivity | HIV testing |
| Chamie et al., 2016 | A hybrid mobile approach for population-wide HIV testing in rural east Africa: An observational study | SEARCH | April 2013 to June 2014 | Cross-sectional | Adults | Kenya, Uganda | Rural/agricultural, Fishing villages, Border areas | Prevention | 2.5 HIV testing volume and positivity | HIV testing |
| Kadede et al., 2016 | Increasing adolescent HIV testing with a hybrid mobile strategy in uganda and Kenya | SEARCH | April 2013 to June 2014 | Cross-sectional | Adolescents and young people | Kenya, Uganda | Rural/agricultural, Fishing villages, Border areas | Prevention, Care and treatment | 2.5 HIV testing volume and positivity | HIV testing, New HIV diagnoses |
| Edwards et al., 2019 | The HIV care continuum among resident and non-resident populations found in venues in East Africa cross-border areas | East Africa Cross Border Integrated Health Study (CBIHS) | June 2016 to February 2017 | Cross-sectional | Adults | Kenya, Rwanda, Tanzania, Uganda | Border areas | Care and treatment | 2.1 People living with HIV who know their HIV status; 2.2 People living with HIV on antiretroviral therapy; 2.3 People living with HIV who have suppressed viral loads | Known HIV positive status, ART coverage, Virally suppressed |
| Larmarange et al., 2018 | The impact of population dynamics on the population HIV care cascade: results from the ANRS 12249 Treatment as Prevention trial in rural KwaZulu-Natal (South Africa) | ANRS 12249 Treatment as Prevention (TasP) trial, ACDIS Cohort | March 2012 to June 2016 | Longitudinal analysis within an RCT | Adults | South Africa | Rural/agricultural, Border area | Care and treatment | 2.1 People living with HIV who know their HIV status; 2.2 People living with HIV on antiretroviral therapy; 2.3 People living with HIV who have suppressed viral loads | Known HIV positive status, Enrolled in care, ART coverage, Virally suppressed |
| Billioux et al., 2017 | Human immunodeficiency virus care cascade among sub-populations in Rakai, Uganda: an observational study. | RCCS | September 2013 to December 2015 | Cross-sectional | Adults | Uganda | Rural/agricultural, Urban/trading center, Fishing villages, Border areas, Transit corridors | Care and treatment | 2.1 People living with HIV who know their HIV status; 2.2 People living with HIV on antiretroviral therapy; 2.3 People living with HIV who have suppressed viral loads | Known HIV status, Enrolled in care, ART initiation, Virally suppressed |
| Dorward et al., 2017 | Factors associated with poor linkage to HIV care in South Africa: Secondary analysis of data from the Thol'impilo trial | Thol’impilo trial | 2012 to 2015 | Longitudinal analysis within an RCT | Adults | South Africa | Rural/agricultural, Urban/trading center | Care and treatment | 2.2 People living with HIV on antiretroviral therapy | Linkage to care |
| Wahome et al., 2022 | Stopping and restarting PrEP and loss to follow-up among PrEP-taking men who have sex with men and transgender women at risk of HIV-1 participating in a prospective cohort study in Kenya | Primary | June 2017 to June 2019 | Cohort | Men who have sex with men and transgender women | Kenya | Urban/trading center, transit corridor | Prevention | 1.11 People who received pre-exposure prophylaxis | PrEP use |
| Koss et al., 2020 | Uptake, engagement, and adherence to pre-exposure prophylaxis offered after population HIV testing in rural Kenya and Uganda: 72-week interim analysis of observational data from the SEARCH study | SEARCH | June 2016 to June 2017 | Longitudinal analysis within an RCT | Adults | Kenya, Uganda | Rural/agricultural, Fishing villages, Border areas | Prevention | 1.11 People who received pre-exposure prophylaxis | PrEP use |
| Shearer et al., 2017 | Citizenship status and engagement in HIV care: An observational cohort study to assess the association between reporting a national ID number and retention in public-sector HIV care in Johannesburg, South Africa | EMR/Program data | January 2008 to February 2015 | Retrospective Cohort | Adults | South Africa | Urban/trading center | Care and treatment | 2.2 People living with HIV on antiretroviral therapy | Retention in care |
| Thorp et al., 2022 | Mobility and ART retention among men in Malawi: a mixed-methods study | ENGAGE and IDEaL trials | August 2021 to January 2022 | Cross-sectional | Men | Malawi | Rural/agricultural | Care and treatment | 2.2 People living with HIV on antiretroviral therapy | Retention in care |
| Beres et al., 2021 | Patterns and Predictors of Incident Return to HIV Care Among Traced, Disengaged Patients in Zambia: Analysis of a Prospective Cohort | Better Information for Health in Zambia (BetterInfo) | August 2013 to January 2018 | Cohort | Adults | Zambia | Rural/agricultural, Urban/trading center, Border area | Care and treatment | 2.2 People living with HIV on antiretroviral therapy | Retention in care |
| Bernardo et al., 2021 | Patterns of mobility and its impact on retention in care among people living with HIV in the Manhica District, Mozambique | Primary | December 2017 to January 2018 | Case-control | Adults | Mozambique | Rural/agricultural, Border area | Care and treatment | 2.2 People living with HIV on antiretroviral therapy | Retention in care |
| Davey et al., 2020 | Exploring the Association Between Mobility and Access to HIV Services Among Female Sex Workers in Zimbabwe | SAPPH-Ire trial | 2016 | Cross-sectional | Female sex workers | Zimbabwe | Urban/trading center, Rural/agricultural, Mining towns, Transit corridor | Prevention, Care and treatment | 2.1 People living with HIV who know their HIV status; 2.2 People living with HIV on antiretroviral therapy; 2.3 People living with HIV who have suppressed viral loads; 2.5 HIV testing volume and positivity; | Retention in care, Known HIV positive status, ART coverage, Virally suppressed, HIV testing |
| Tomita et al., 2019 | Sociobehavioral and community predictors of unsuppressed HIV viral load: Multilevel results from a hyperendemic rural South African population | ACDIS Cohort | 2011 to 2014 | Cohort | Women | South Africa | Rural/agricultural, Border area | Care and treatment | 2.3 People living with HIV who have suppressed viral loads | Viral suppression |
| Senteza et al., 2023 | Virological non-suppression among adult males attending HIV care services in the fishing communities in Bulisa district, Uganda | EMR/Program data | January 2019 to January 2020 | Cross-sectional | Men | Uganda | Fishing villages | Care and treatment | 2.3 People living with HIV who have suppressed viral loads | Viral suppression |
| Plymoth et al., 2020 | Socio-economic condition and lack of virological suppression among adults and adolescents receiving antiretroviral therapy in Ethiopia | Primary | October to December 2018 | Case-control | Adults | Ethiopia | Urban/trading center, transit corridor | Care and treatment | 2.3 People living with HIV who have suppressed viral loads | Viral suppression |
| Grabowski et al., 2021 | Prevalence and Predictors of Persistent Human Immunodeficiency Virus Viremia and Viral Rebound after Universal Test and Treat: A Population-Based Study | RCCS | November 2011 to August 2017 | Cohort | Adults | Uganda | Rural/agricultural, Urban/trading center, Fishing villages, Border areas, Transit corridors | Care and treatment | 2.3 People living with HIV who have suppressed viral loads | Viral suppression |
| Brophy et al., 2021 | Prevalence of Untreated HIV and Associated Risk Behaviors Among the Sexual Partners of Recent Migrants and Long-term Residents in Rakai, Uganda | RCCS | April 1999 to September 2016 | Cohort | Adults | Uganda | Rural/agricultural, Urban/trading center, Fishing villages, Border areas, Transit corridors | Epidemiology, Prevention | HIV Prevalence; 1.14 Condom use at last high risk sex; 1.12 Prevalence of male circumcision | VMMC, condom use, HIV serostatus |

## Table S6. Study-specific age and gender stratifications

| **Reference** | **Title** | **Age-stratified mobility measure** | **Gender-stratified mobility measure** | **Age-stratified relationship** | **Gender-stratified relationship** |
| --- | --- | --- | --- | --- | --- |
| Baisley et al., 2018 | High HIV incidence and low uptake of HIV prevention services: The context of risk for young male adults prior to DREAMS in rural KwaZulu-Natal, South Africa | Yes | N/A | N/A | N/A |
| Nhampossa et al., 2021 | The impact of the caregiver mobility on child HIV care in the Manhica District, Southern Mozambique: A clinical based study | Yes | Yes | N/A | No |
| Janse Van Rensburg et al., 2021 | Healthcare without borders: A cross-sectional study of immigrant and nonimmigrant children admitted to a large public sector hospital in the Gauteng Province of South Africa | Yes | No | N/A | No |
| Odimegwu et al., 2022 | A multilevel mixed effect analysis of neighbourhood and individual level determinants of risky sexual behaviour among young people in South Africa. | No | Yes | N/A | Yes |
| Bwambale et al., 2021 | Utilisation of sexual and reproductive health services among street children and young adults in Kampala, Uganda: does migration matter? | No | No | N/A | No |
| Kadede et al., 2016 | Increasing adolescent HIV testing with a hybrid mobile strategy in uganda and Kenya | No | No | N/A | No |
| Low et al., 2019-B | Correlates of HIV infection in adolescent girls and young women in Lesotho: results from a population-based survey | Yes | N/A | N/A | N/A |
| Chimbindi et al., 2018 | Persistently high incidence of HIV and poor service uptake in adolescent girls and young women in rural KwaZulu-Natal, South Africa prior to DREAMS | Yes | N/A | N/A | N/A |
| Mthiyane et al., 2022 | The association of exposure to DREAMS on sexually acquiring or transmitting HIV amongst adolescent girls and young women living in rural South Africa | No | N/A | N/A | N/A |
| Agadjanian et al., 2021 | Men's migration and women's mortality in rural Mozambique | No | N/A | No | N/A |
| Bahemuka et al., 2023 | Factors Associated with Short and Long Term Mobility and HIV Risk of Women Living in Fishing Communities Around Lake Victoria in Kenya, Tanzania, and Uganda: A Cross Sectional Survey | Yes | No | No | N/A |
| Lee et al., 2023 | Condom, modern contraceptive, and dual method use are associated with HIV status and relationship concurrency in a context of high mobility: A cross-sectional study of women of reproductive age in rural Kenya and Uganda, 2016 | Yes | N/A | No | N/A |
| Chawhanda et al., 2023 | Factors associated with access to condoms and HIV services among women in high migration communities in six Southern African countries | No | N/A | No | N/A |
| Dzomba et al., 2019 | Effect of ART scale-up and female migration intensity on risk of HIV acquisition: results from a population-based cohort in KwaZulu-Natal, South Africa | Yes | N/A | No | N/A |
| Tomita et al., 2019 | Sociobehavioral and community predictors of unsuppressed HIV viral load: Multilevel results from a hyperendemic rural South African population | No | N/A | No | NA |
| Rosenberg et al., 2018 | Individual, Partner, and Couple Predictors of HIV Infection among Pregnant Women in Malawi: A Case-Control Study | No | N/A | No | NA |
| Fennell et al., 2023 | The impact of free antiretroviral therapy for pregnant non-citizens and their infants in Botswana | No | N/A | No | N/A |
| Odayar et al., 2023 | Mobility during the post-partum period and viraemia in women living with HIV in South Africa | Yes | N/A | No | N/A |
| Reidy et al., 2018 | Engagement in care and infant HIV testing among lost to follow-up option B+ patients | No | N/A | No | NA |
| Wahome et al., 2022 | Stopping and restarting PrEP and loss to follow-up among PrEP-taking men who have sex with men and transgender women at risk of HIV-1 participating in a prospective cohort study in Kenya | No | No | No | No |
| Senteza et al., 2023 | Virological non-suppression among adult males attending HIV care services in the fishing communities in Bulisa district, Uganda | No | N/A | No | NA |
| Slabbert et al., 2017 | Sexual and reproductive health outcomes among female sex workers in Johannesburg and Pretoria, South Africa: Recommendations for public health programmes | No | N/A | No | NA |
| Shaw et al., 2023 | Geographical Associations of HIV Prevalence in Female Sex Workers From Nairobi, Kenya (2014-2017) | Yes | N/A | No | NA |
| Vandormael et al., 2020 | HIV incidence declines in a rural South African population: a G-imputation approach for inference | No | No | No | Yes |
| Tomita et al., 2017 | Social Disequilibrium and the Risk of HIV Acquisition: A Multilevel Study in Rural KwaZulu-Natal Province, South Africa | No | Yes | No | No |
| Nyabuti et al., 2021 | Characteristics of HIV seroconverters in the setting of universal test and treat: Results from the SEARCH trial in rural Uganda and Kenya | No | Yes | no | Yes |
| Psaki et al., 2022 | What are we learning about HIV testing in informal settlements in KwaZulu-Natal, South Africa? Results from a randomized controlled trial | No | Yes | no | Yes |
| Psaki et al., 2022 | What are we learning about HIV testing in informal settlements in KwaZulu-Natal, South Africa? Results from a randomized controlled trial | No | Yes | No | Yes |
| Nakiire et al., 2020 | Factors Associated with Utilisation of Couple HIV Counselling and Testing Among HIV-Positive Adults in Kyoga Fishing Community Uganda, May 2017: Cross Sectional Study | No | No | No | No |
| Shearer et al., 2017 | Citizenship status and engagement in HIV care: An observational cohort study to assess the association between reporting a national ID number and retention in public-sector HIV care in Johannesburg, South Africa | Yes | Yes | No | No |
| Plymoth et al., 2020 | Socio-economic condition and lack of virological suppression among adults and adolescents receiving antiretroviral therapy in Ethiopia | No | No | No | No |
| Petersen et al., 2021 | Geographic mobility and time to seeking care among people with TB in Limpopo, South Africa | Yes | Yes | No (but wasn't the primary association studied) | No (but wasn't the primary association studied) |
| Kim et al., 2020 | Migration and first-year maternal mortality among HIV-positive postpartum women: A population-based longitudinal study in rural South Africa | No | N/A | No | N/A |
| Goodman et al., 2016 | Child-street migration among HIV-affected families in Kenya: a mediation analysis from cross-sectional data. | No | N/A | No | No |
| Thorp et al., 2022 | Mobility and ART retention among men in Malawi: a mixed-methods study | No | N/A | No | N/A |
| Dememew et al., 2020 | The yield of community-based tuberculosis and HIV among key populations in hotspot settings of Ethiopia: A cross-sectional implementation study | No | No | No | N/A |
| Gorin et al., 2023 | Mobility and HIV vulnerabilities among female sex workers in Guinea-Bissau: findings from an integrated bio-behavioral survey | No | No | No | N/A |
| Davey et al., 2019 | Mobility and sex work: why, where, when? A typology of female-sex-worker mobility in Zimbabwe | Yes | N/A | No | N/A |
| Kiyingi et al., 2023 | Predictors of mobility among women engaged in commercial sex work in Uganda using generalized estimating equations model | No | N/A | No | N/A |
| Coetzee et al., 2017 | Cross-sectional study of female sex workers in Soweto, South Africa: Factors associated with HIV infection | No | N/A | No | N/A |
| Davey et al., 2020 | Exploring the Association Between Mobility and Access to HIV Services Among Female Sex Workers in Zimbabwe | No | N/A | No | N/A |
| Camlin et al., 2017 | High mobility and HIV prevalence among female market traders in East Africa in 2014 | No | N/A | No | N/A |
| Camlin et al., 2017 | High mobility and HIV prevalence among female market traders in East Africa in 2014 | No | N/A | No | N/A |
| Camlin et al., 2017 | High mobility and HIV prevalence among female market traders in East Africa in 2014 | No | N/A | No | N/A |
| Marukutira et al., 2019-A | Clinical outcomes of a cohort of migrants and citizens living with human immunodeficiency virus in Botswana: Implications for Joint United Nation Program on HIV and AIDS 90-90-90 targets | Yes | Yes | No | No |
| Murnane et al., 2022 | Distinct forms of migration and mobility are differentially associated with HIV treatment adherence | No | Yes | No | Yes |
| Murnane et al., 2022 | Distinct forms of migration and mobility are differentially associated with HIV treatment adherence | No | Yes | No | Yes |
| Etoori et al., 2020 | Outcomes After Being Lost to Follow-up Differ for Pregnant and Postpartum Women When Compared With the General HIV Treatment Population in Rural South Africa | No | Yes | No | Yes |
| Ambia et al., 2019 | Outcomes of patients lost to follow-up after antiretroviral therapy initiation in rural north-eastern South Africa | No | No | No | No |
| Onoya et al., 2021 | Understanding the Reasons for Deferring ART Among Patients Diagnosed Under the Same‐Day‐ART Policy in Johannesburg, South Africa | No | Yes | No | No |
| Olawore et al., 2018 | Migration and risk of HIV acquisition in Rakai, Uganda: a population-based cohort study | Yes | Yes | No | Yes |
| Dobra et al., 2017 | Space-time migration patterns and risk of HIV acquisition in rural South Africa | Yes | Yes | No | Yes |
| Dobra et al., 2019 | A method for statistical analysis of repeated residential movements to link human mobility and HIV acquisition | Yes | Yes | No | No |
| Low et al., 2021 | Migration in Namibia and its association with HIV acquisition and treatment outcomes | No | Yes | No | Yes |
| Camlin et al., 2019 | Gendered dimensions of population mobility associated with HIV across three epidemics in rural Eastern Africa | No | Yes | No | Yes |
| Camlin et al., 2019 | Gendered dimensions of population mobility associated with HIV across three epidemics in rural Eastern Africa | No | Yes | No | Yes |
| Dzomba et al., 2022 | Predictors of migration in an HIV hyper-endemic rural South African community: evidence from a population-based cohort (2005-2017) | Yes | Yes | No | Yes |
| Ginsburg et al., 2021 | Internal migration and health in South Africa: determinants of healthcare utilisation in a young adult cohort | Yes | Yes | No | No |
| Low et al., 2019-A | Association between severe drought and HIV prevention and care behaviors in Lesotho: A population-based survey 2016-2017 | No | No | Yes | No |
| Bulstra et al., 2020 | Mapping and characterising areas with high levels of HIV transmission in sub-Saharan Africa: A geospatial analysis of national survey data | Yes | Yes | No | No |
| Grabowski et al., 2020 | Migration, hotspots, and dispersal of HIV infection in Rakai, Uganda | Yes | Yes | Yes | Yes |
| Marukutira et al., 2019-B | Comparison of knowledge of HIV status and treatment coverage between non-citizens and citizens: Botswana Combination Prevention Project (BCPP) | Yes | Yes | No | No |
| Correa-Agudelo et al., 2021 | Associated health and social determinants of mobile populations across HIV epidemic gradients in Southern Africa. | No | No | No | No |
| Floyd et al., 2020 | HIV testing and treatment coverage achieved after 4 years across 14 urban and peri-urban communities in Zambia and South Africa: An analysis of findings from the HPTN 071 (PopART) trial | Yes | Yes | No | No |
| Chamie et al., 2016 | A hybrid mobile approach for population-wide HIV testing in rural east Africa: An observational study | No | No | No | No |
| Edwards et al., 2019 | The HIV care continuum among resident and non-resident populations found in venues in East Africa cross-border areas | Yes | Yes | No | No |
| Larmarange et al., 2018 | The impact of population dynamics on the population HIV care cascade: results from the ANRS 12249 Treatment as Prevention trial in rural KwaZulu-Natal (South Africa) | No | No | No | No |
| Billioux et al., 2017 | Human immunodeficiency virus care cascade among sub-populations in Rakai, Uganda: an observational study. | No | No | No | No |
| Dorward et al., 2017 | Factors associated with poor linkage to HIV care in South Africa: Secondary analysis of data from the Thol'impilo trial | No | No | No | No |
| Koss et al., 2020 | Uptake, engagement, and adherence to pre-exposure prophylaxis offered after population HIV testing in rural Kenya and Uganda: 72-week interim analysis of observational data from the SEARCH study | Yes | No | No | Yes |
| Beres et al., 2021 | Patterns and Predictors of Incident Return to HIV Care Among Traced, Disengaged Patients in Zambia: Analysis of a Prospective Cohort | No | No | No | No |
| Bernardo et al., 2021 | Patterns of mobility and its impact on retention in care among people living with HIV in the Manhica District, Mozambique | No | No | No | No |
| Grabowski et al., 2021 | Prevalence and Predictors of Persistent Human Immunodeficiency Virus Viremia and Viral Rebound after Universal Test and Treat: A Population-Based Study | No | No | No | Yes |
| Brophy et al., 2021 | Prevalence of Untreated HIV and Associated Risk Behaviors Among the Sexual Partners of Recent Migrants and Long-term Residents in Rakai, Uganda | No | Yes | No | Yes |
